# The *Pseudomonas aeruginosa* DedA protein PA4029 is an undecaprenyl phosphate flippase important for polymyxin resistance

**DOI:** 10.1128/mbio.02408-25

**Published:** 2026-01-12

**Authors:** Davide Sposato, Yi Wang, Xinye Zhang, Ludovica Rossi, Stefania De Chiara, Flaviana Di Lorenzo, Livia Leoni, Giordano Rampioni, Paolo Visca, Jani R. Bolla, Francesco Imperi

**Affiliations:** 1 Department of Science, University Roma Tre 164756, Rome, Italy; 2 Department of Biology, University of Oxford 6396, Oxford, United Kingdom; 3 Department of Chemical Sciences, University of Naples Federico II 9307, Naples, Italy; 4 CEINGE-Biotecnologie Avanzate Franco Salvatore 18311 , Naples, Italy; 5 IRCCS Fondazione Santa Lucia 9362 , Rome, Italy; 6 NBFC, National Biodiversity Future Center, Palermo, Italy; Case Western Reserve University School of Medicine, Cleveland, Ohio, USA

**Keywords:** aminoarabinose, colistin, DedA, DUF368, native mass spectrometry, peptidoglycan, transporter, undecaprenyl phosphate

## Abstract

Undecaprenyl phosphate (C55-P) is a critical lipid carrier required for the transport of cell envelope precursors across the cytoplasmic membrane in bacteria. Recent studies have identified proteins of the DedA family and DUF368 domain family as C55-P flippases in both Gram-positive and Gram-negative organisms. However, their roles remain undefined in many clinically relevant pathogens. Here, we screened for DedA and DUF368 proteins in *Pseudomonas aeruginosa* and assessed their functional importance. We show that PA4029, a DedA family membrane protein, is involved in C55-P recycling. Deletion of PA4029 sensitizes cells to fosmidomycin and limits the emergence of spontaneous colistin-resistant mutants. Using native mass spectrometry, we demonstrate that PA4029 binds C55-P with high affinity and selectivity over membrane phospholipids, and that this interaction is disrupted by the C55-P targeting antibiotic amphomycin. We also show that a DUF368 protein, found in some *Pseudomonas* species lacking PA4029 orthologs, can functionally substitute for PA4029 in *P. aeruginosa*, suggesting divergent strategies for C55-P recycling in this genus. Together, these findings position PA4029 within the conserved DedA-mediated lipid carrier pathway and highlight its importance for cell envelope homeostasis and antibiotic resistance in *P. aeruginosa*.

**IMPORTANCE:**

Bacteria use lipid carrier undecaprenyl phosphate (C55-P) to build and maintain their cell envelope, which is necessary for survival and is the target of many antibiotics. Recent studies have implicated DedA family proteins in C55-P transport, but how these proteins function in important pathogens like *Pseudomonas aeruginosa* remains uncharacterized. In this work, we uncover a specific DedA protein, PA4029, and show its involvement in C55-P recycling and importance for bacteria’s ability to develop resistance to the last-resort antibiotic colistin. These findings extend the relevance of DedA-mediated lipid transport to one of the most dreaded human pathogens.

## INTRODUCTION

The bacterial cell wall is a defining feature of most prokaryotic organisms and plays a crucial role in maintaining cellular integrity, shape, and viability. The cell wall allows bacteria to withstand internal turgor pressure and provides protection against environmental stressors. The structural rigidity of the cell wall is largely attributed to peptidoglycan, an essential, mesh-like polymer composed of alternating units of N-acetylglucosamine and N-acetylmuramic acid, crosslinked via short peptide stems (1, 2). The peptidoglycan is the target of many clinically relevant antibiotics, including β-lactams, glycopeptides, and fosfomycin (3).

The biosynthesis of peptidoglycan is a complex process that involves (i) synthesis of precursors in the cytoplasm, (ii) membrane-associated steps where precursors are assembled onto a lipid carrier, and (iii) polymerization and crosslinking of precursors on the outer side of the cytoplasmic membrane (CM) (2). A critical component of the membrane-associated steps is undecaprenyl phosphate (C55-P), a long-chain polyisoprenoid lipid that serves as the carrier for peptidoglycan monomers (4). The disaccharide-pentapeptide precursor is assembled and linked to C55-P to form the so-called lipid II, which is flipped to the outer leaflet of the CM by the membrane protein MurJ (5) and finally polymerized into the peptidoglycan meshwork by transglycosylase and transpeptidase enzymes (6). Monomer release generates C55-PP, which is dephosphorylated to C55-P and flipped to the inner leaflet of the CM to complete the cycle (7). Besides peptidoglycan precursors, C55-P also mediates the transport of monomers for other glycopolymers, such as O-antigens, teichoic acids, and extracellular polysaccharides, as well as sugars used in the glycosylation of lipid A of lipopolysaccharides or surface proteins (8). These processes depend on specific transporters that flip C55-P-(oligo)saccharide intermediates toward the outer leaflet of the CM (9–11).

Given its central role in the biosynthesis of peptidoglycan and other cell envelope structures, C55-P(P) is an essential resource that must be efficiently recycled. Therefore, it is not surprising that some antibiotics exert their activity by sequestering intermediates of the lipid II cycle, such as lipid II (nisin), C55-PP (bacitracin), or C55-P (amphomycin) (12, 13). These are, however, only active against Gram-positive bacteria, as their size prevents diffusion across the outer membrane of Gram-negatives (12). Moreover, several enzymes involved in C55-P metabolism have been investigated as targets for novel antibacterial drugs, such as the C55-P synthase UppS (14), C55-PP phosphatase BacA/UppP (15), and lipid II flippase MurJ (16).

While the transporter of lipid II was identified more than a decade ago (5, 17), the retrograde flipping of C55-P across the CM remained elusive for years. Recently, two independent studies provided compelling evidence that proteins belonging to the DedA and DUF368 families are responsible for flipping back C55-P in representatives of both Gram-negative (*Vibrio cholerae*) and Gram-positive bacteria (*Bacillus subtilis* and *Staphylococcus aureus*) (18, 19). DedA proteins are highly conserved and nearly ubiquitous across bacteria (20), while DUF368 proteins are present in less than 30% of bacterial species (18). Most bacteria have multiple DedA proteins (e.g., eight in *Escherichia coli* and six in *B. subtilis*), but only some of them exhibit C55-P flippase activity or are important for cell viability (19–22), implying that DedA proteins can also perform additional functions. For instance, some bacterial DedA proteins resemble eukaryotic phospholipid scramblases (20) and, accordingly, the DedA protein PetA of *B. subtilis* functions as a phosphatidylethanolamine (PE) transporter (23). Moreover, some DedA proteins have been implicated in resistance to cationic compounds and/or polymyxins in various bacteria (24–28), although the underlying molecular mechanisms remained unclear.

In this study, we investigated the DedA proteins of the Gram-negative human pathogen *Pseudomonas aeruginosa*, where their functions have not yet been explored. By integrating genetic, phenotypic, biochemical, and biophysical assays, we provide evidence supporting PA4029 as the main *P. aeruginosa* DedA protein involved in flipping back C55-P, as its inactivation results in defects consistent with impaired C55-P recycling, and the purified protein specifically binds C55-P. Notably, the lack of PA4029 compromises colistin resistance and limits the emergence of spontaneous colistin-resistant mutants. While PA4029 is conserved across most *Pseudomonas* species, certain pseudomonads instead have a DUF368 protein which can functionally replace PA4029 in *P. aeruginosa*, suggesting divergent evolutionary paths for C55-P flippases within the *Pseudomonas* genus.

## RESULTS

### *P. aeruginosa* has six DedA proteins

To perform a phylogenetic analysis of the bacterial DedA proteins, Todor et al. recently searched for DedA homologs in the proteomes derived from 6,000 bacterial genomes, by using the PF09335.14 motif characteristic of DedA family proteins as the query. This led to the identification of more than 16,000 bacterial DedA proteins, five of which were identified in *P. aeruginosa* (20). BLAST analysis revealed that these proteins correspond to the genes/proteins PA1209, PA4011, PA4029, PA5244, and PA5517 in the reference strain *P. aeruginosa* PAO1, all described as hypothetical proteins in the *Pseudomonas* Genome Database (https://pseudomonas.com/). Notably, one of these proteins (PA5517) is not annotated as a member of the PF09335 family (https://pseudomonas.com/) (Table 1).

**TABLE 1:** DedA-like proteins identified in *P. aeruginosa*

*P. aeruginosa* PAO1 DedA-like protein (aa)^*a*^	Pfam	*P. aeruginosa* genomes with ortholog^*b*^	*Pseudomonas* genomes (other than *P. aeruginosa*) with ortholog^*c*^
PA1209 (311)	PF09335 and PF00581	297/297 (100%)	10/236 (4.2%)
PA2752 (147)	PF09335	297/297 (100%)	209/236 (88.6%)
PA4011 (437)	PF09335 and PF01569	296/297 (99.7%)^*d*^	235/236 (99.6%)
PA4029 (221)	PF09335	297/297 (100%)	201/236 (85.2%)
PA5244 (196)	PF09335	297/297 (100%)	179/236 (75.8%)
PA5517 (194)		294/297 (99%)^*e*^	4/236 (1.7%)

^*a*^ According to the results of Todor et al. (20) and BLASTP analysis (see Table S1).

^*b*^ Putative orthologs were retrieved from 297 genomes according to the *Pseudomonas* Ortholog Groups of the *Pseudomonas* Genome Database (https://pseudomonas.com) and individually checked.

^*c*^ Putative orthologs were retrieved from 236 genomes according to the *Pseudomonas* Ortholog Groups of the *Pseudomonas* Genome Database (https://pseudomonas.com/).

^*d*^ The ortholog of the *P. aeruginosa* strain MRW44.1 (A542_RS0128425) is annotated as a pseudogene.

^*e*^ The orthologs of the *P. aeruginosa* strains BWHPSA037 (Q042_RS13290) and AZPAE15065 (NT81_RS08910) are annotated as pseudogenes, while the ortholog appears to be absent from the strain PA7.

To verify whether other DedA-like proteins are present in *P. aeruginosa*, we performed a BLAST analysis to search the proteome of *P. aeruginosa* PAO1 for additional homologs of (i) the eight DedA proteins of *E. coli* (22), (ii) the five DedA proteins identified in *P. aeruginosa* by Todor et al. (20), and (iii) DedA proteins recently demonstrated to function as lipid flippases in other bacteria (18, 19, 23). The results of this analysis, reported in Table S1, revealed that another member of the DedA family is present in *P. aeruginosa* PAO1 (PA2752) (Table 1). Orthologs of the six *P. aeruginosa* PAO1 *dedA*-like genes are well conserved in almost all *P. aeruginosa* strains, while other *Pseudomonas* species mainly harbor orthologs of PA2752, PA4011, PA4029, and PA5244, with PA1209 and PA5517 being present in only a few species (Table 1; Table S2).

The putative 3D structure and transmembrane helices (TMHs) of the *P. aeruginosa* DedA proteins are shown in Fig. S1. PA2752, PA4029, PA5244, and PA5517 only contain the DedA-like domain, whilst PA1209 and PA4011 are predicted to be bifunctional proteins with an N-terminal DedA-like domain and a C-terminal rhodanese-like (PF00581) or PAP2-like (PF01569) domain, respectively (Table 1 and Fig. S1). Bacterial rhodaneses catalyze sulfur transfer reactions and are generally involved in cyanide detoxification and/or sulfur metabolism (29). The PAP2 family contains both soluble and integral membrane proteins, including nonspecific acid phosphatases, lipid phosphatases, and other uncharacterized enzymes. Some bacterial membrane PAP2 proteins have been reported to play a role in the metabolism of glycerophospholipids, C55-P, or lipopolysaccharides (8, 30).

### Individual DedA deletions do not compromise growth or cell envelope integrity

**Fig 1:**
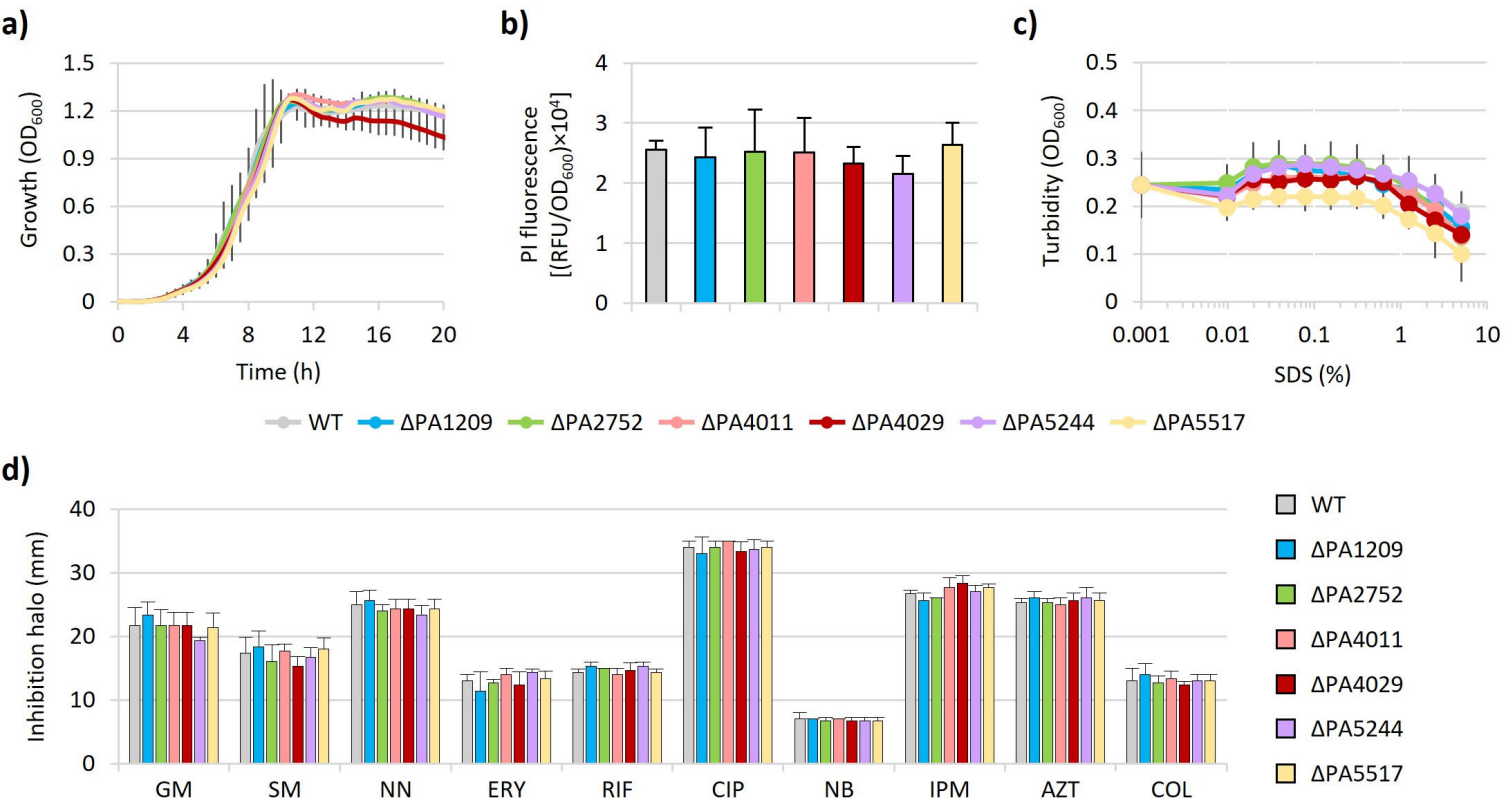
Effect of DedA proteins on *P. aeruginosa* growth and cell envelope integrity. (**a**) Growth curves of *P. aeruginosa* PAO1 (WT) and isogenic mutants in *dedA*-like genes cultured in MH. (**b**) Permeability to PI, measured as relative fluorescence units (RFU) normalized to the OD_600_ of the bacterial suspension, of WT and *dedA* mutants. (**c**) Sensitivity to the lytic effect of different concentrations of SDS (0%–5%), measured as a decrease in cell suspension turbidity (OD_600_), of WT and *dedA* mutants. (**d**) Antibiotic sensitivity profile of WT and *dedA* mutants determined through the Kirby-Bauer disk diffusion test on MH agar plates. GM, gentamycin; SM, streptomycin; NN, tobramycin; ERY, erythromycin; RIF, rifampicin; CIP, ciprofloxacin; NB, novobiocin; IPM, imipenem; AZT, aztreonam; COL, colistin. Values are the mean (±standard deviation) of three independent experiments. No statistically significant differences were observed between the WT and each deletion mutant (*P* > 0.05; one-way analysis of variance [ANOVA], Kruskal-Wallis test).

To investigate the role of DedA proteins in *P. aeruginosa*, we generated six deletion mutants in the reference strain PAO1, each lacking one *dedA* gene. These mutants were preliminarily tested for the ability to grow in a rich medium. As shown in Fig. 1a, the growth curves of the mutants were comparable to that of the parental strain, implying that individual DedA proteins are not required for *P. aeruginosa* cell viability and growth.

To assess whether the absence of DedA proteins might affect cell envelope functionality in *P. aeruginosa*, we first measured the uptake of propidium iodide (PI). This fluorescent probe is commonly used to assess membrane integrity, as it only enters cells with damaged membranes and its fluorescence increases upon binding to DNA (31, 32). None of the *dedA* mutants showed any increase in fluorescence with respect to the parental strain (Fig. 1b), ruling out relevant defects in the membrane permeability barrier. Then, we evaluated the sensitivity of the mutants to the lytic effect of the detergent SDS, as an indicator of cell envelope damage (33, 34). Also in this case, deletion of any individual *dedA* gene did not result in increased susceptibility to SDS (Fig. 1c). Finally, to further verify the functionality of the cell envelope as a permeability barrier, we also performed a Kirby-Bauer disk diffusion assay to assess the resistance profile of *dedA* mutants to 10 antibiotics belonging to different classes and having different mechanisms of action. Once again, no differences were observed between *dedA* mutants and the parental strain (Fig. 1d), indicating that the absence of each individual DedA protein does not promote antibiotic entry and, thus, confirming that the permeability barrier of the cell envelope is not compromised. Overall, these assays rule out that individual DedA proteins are essential for cell viability and cell envelope integrity in *P. aeruginosa*.

### PA4029 is likely involved in undecaprenyl phosphate recycling

**Fig 2:**
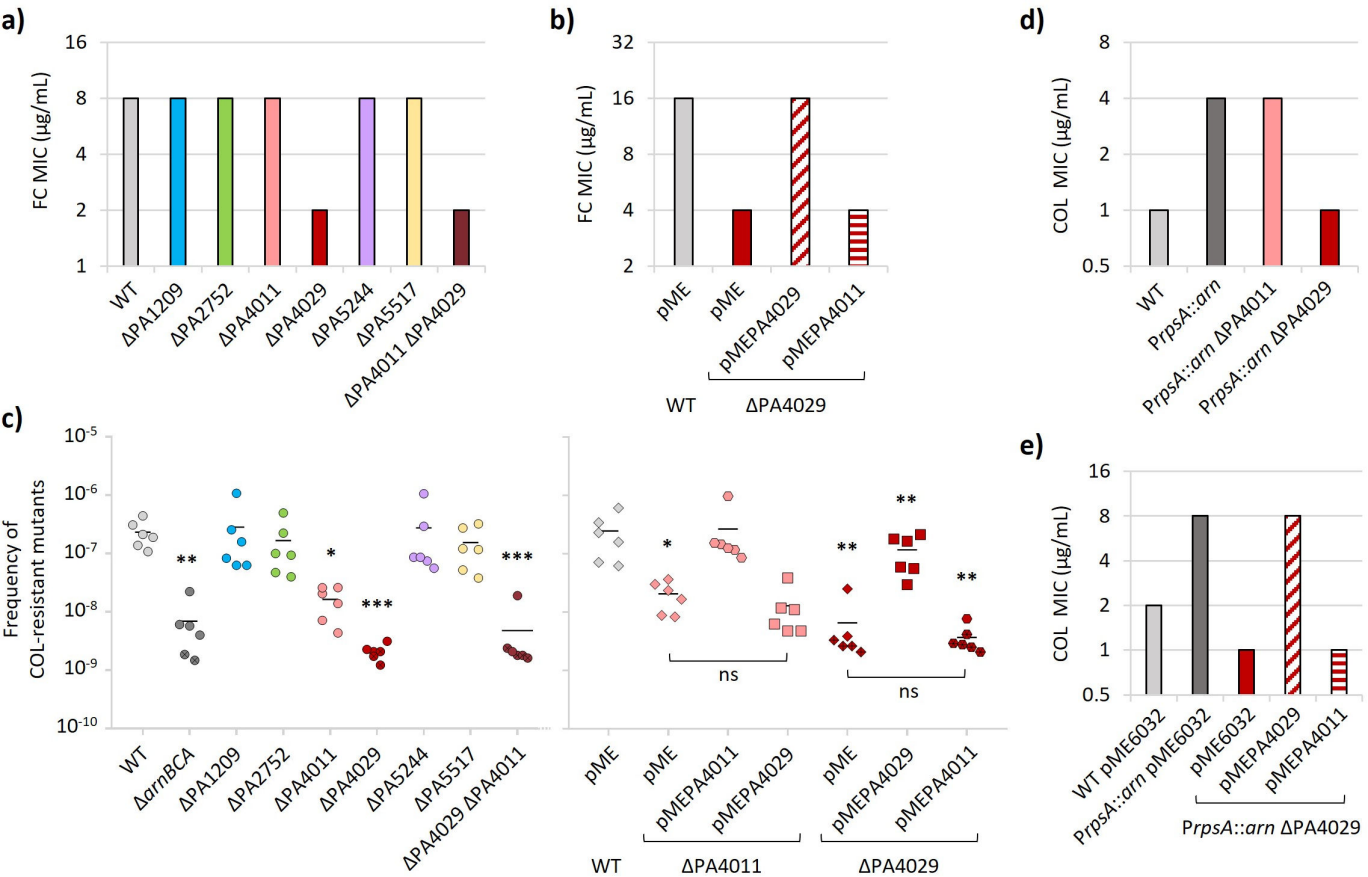
Role of PA4029 in C55-P recycling *in vivo*. (**a**) Fosmidomycin (FC) MIC for *P. aeruginosa* PAO1 (WT), the single mutants in *dedA*-like genes, and the double mutant ΔPA4011 ΔPA4029. (**b**) FC MIC for the WT and ΔPA4029 strains carrying the empty plasmid pME6032 (pME) or its derivatives for ectopic expression of PA4011 or PA4029. (**c**) Frequency of colistin-resistant spontaneous mutants for the WT strain and *dedA* mutants (left panel), and the mutants ∆PA4011 and ∆PA4029 ectopically expressing PA4011 or PA4029 (right panel). The ∆*arnBCA* mutant (left panel) and the strains carrying the empty plasmid pME (right panel) were used as controls. Each symbol corresponds to an individual experiment. Crossed symbols correspond to half the limit of detection of the individual experiment in which no colonies appeared on selective plates. (**d**) Colistin (COL) MIC for the WT, the recombinant strain P*rpsA::arn,* and its ΔPA4011 and ΔPA4029 isogenic deletion mutants. (**e**) Colistin MIC for the recombinant strain P*rpsA::arn* and its deletion mutants ∆PA4011 and ∆PA4029 carrying the empty plasmid pME or its derivatives for ectopic expression of PA4011 or PA4029. Values in panels a, b, c, and d are the mode of at least three independent experiments. Asterisks in panel c indicate a statistically significant difference with respect to WT or WT pME (*P < 0.05, **P < 0.01, *** *P <* 0.001; ns, not significant; ANOVA, Kruskal-Wallis test).

Some DedA proteins have been proposed to act as flippases in the recycling of C55-P in *V. cholerae*, *B. subtilis*, and *S. aureus* (18, 19). To verify whether any *P. aeruginosa* DedA protein might perform a similar function, we evaluated the sensitivity of *dedA* mutants to the antibiotic fosmidomycin, which inhibits the 1-deoxy-D-xylulose-5-phosphate reductoisomerase enzyme that produces isopentenyl diphosphate, a precursor of C55-P in the *de novo* synthesis pathway (8, 35). Previous studies in *E. coli* showed that mutants impaired in C55-P recycling are more susceptible to fosmidomycin (36), confirming that fosmidomycin sensitivity can be used as a proxy to assess defects in C55-P recycling in Gram-negative bacteria. As shown in Fig. 2a, the mutant lacking PA4029 displayed a fourfold decrease in fosmidomycin MIC with respect to the parental strain, while the deletion of the other *dedA* genes did not affect fosmidomycin susceptibility. Complementation of the ΔPA4029 mutant through ectopic expression of PA4029 restored fosmidomycin resistance to the levels of the parental strain carrying the empty plasmid (Fig. 2b), confirming that the increase in fosmidomycin sensitivity was specifically caused by the lack of PA4029. This result points to PA4029 as the main DedA protein involved in C55-P recycling in *P. aeruginosa*.

Since C55-P is also used as a carrier for flipping aminoarabinose (L-Ara4N) across the CM (9), whose addition to lipid A is responsible for colistin resistance in *P. aeruginosa* (37–39), we hypothesized that defects in C55-P recycling could compromise the ability to acquire colistin resistance. No differences were observed in colistin MIC between the six *dedA* mutants and the parental strain (1 µg/mL for all strains). This was, however, expected, as the lipid A of colistin-sensitive *P. aeruginosa* isolates (including PAO1) is not modified with L-Ara4N (40) and, accordingly, mutants defective in L-Ara4N biosynthesis show the same colistin MIC as their colistin-sensitive parental strains (37). Thus, we performed a mutant selection assay on agar plates supplemented with colistin (10× MIC) to assess whether the lack of DedA proteins can affect the emergence of colistin resistance, which in *P. aeruginosa* is generally associated with mutations inducing lipid A aminoarabinosylation (38, 39). Notably, the ΔPA4029 and ΔPA4011 mutants displayed a frequency of colistin-resistant spontaneous mutants significantly lower than the parental strain, comparable to the L-Ara4N-deficient mutant ∆*arnBCA* used as a control (Fig. 2c) (37). In contrast, no significant differences were observed between the other *dedA* mutants and PAO1 (Fig. 2c). Complementation assays confirmed that the ectopic expression of PA4029 or PA4011 in the corresponding mutants effectively restored their ability to evolve colistin resistance (Fig. 2c). To verify whether the reduced ability of the ΔPA4029 and ΔPA4011 mutants to develop colistin resistance was specifically due to impaired lipid A aminoarabinosylation, we individually deleted the PA4011 or PA4029 gene in a recombinant strain (PAO1 P*rpsA::arn*) which has higher colistin resistance because of constitutive expression of the *arn* operon and the consequent lipid A modification with L-Ara4N (40). Remarkably, deletion of PA4029 in PAO1 P*rpsA::arn* resensitized this strain to colistin, restoring susceptibility comparable to the wild-type strain PAO1 (Fig. 2d), and reduced the aminoarabinosylated lipid A species to undetectable levels (Fig. S2). Conversely, PA4011 deletion had no effect on colistin resistance in the recombinant strain (Fig. 2d). Again, ectopic expression of PA4029 restored colistin resistance to the level of PAO1 P*rpsA::arn* carrying the empty vector (Fig. 2e). Although indirect, these results further support the hypothesis that PA4029 is involved in the flipping of C55-P during the recycling process.

In contrast, the function of PA4011 remains elusive. As discussed above, its absence impaired the acquisition of colistin resistance but did not affect the colistin resistance level conferred by lipid A aminoarabinosylation. Notably, the deletion of PA4011 in the ΔPA4029 mutant did not exacerbate fosmidomycin sensitivity (Fig. 2a), and plasmid-mediated overexpression of PA4011 in PA4029-deficient cells failed to rescue the defects associated with PA4029 deficiency (Fig. 2b and e). Altogether, this body of evidence strongly suggests that the two proteins fulfill distinct roles in *P. aeruginosa*, implying that PA4011 is likely not involved in the flip-back of C55-P.

### Purified PA4029 binds C55-P

To investigate whether PA4029 interacts directly with C55-P, we purified *P. aeruginosa* PA4029. The protein was then analyzed by native mass spectrometry (MS) in a buffer containing 200 mM ammonium acetate (pH 7.0) and 0.05% LDAO. The resulting spectrum revealed two main charge state distributions corresponding to monomeric (25,477.93 ± 0.1 Da) and dimeric (50,958.21 ± 0.23 Da) forms of PA4029 (Fig. S3). Notably, the observed monomeric mass was ~27 Da higher than the theoretical sequence mass (25,451.12 Da), a difference consistent with formylation of the N-terminal methionine, a modification also observed in other bacterial DedA proteins, such as UptA from *B. subtilis* (41).

**Fig 3:**
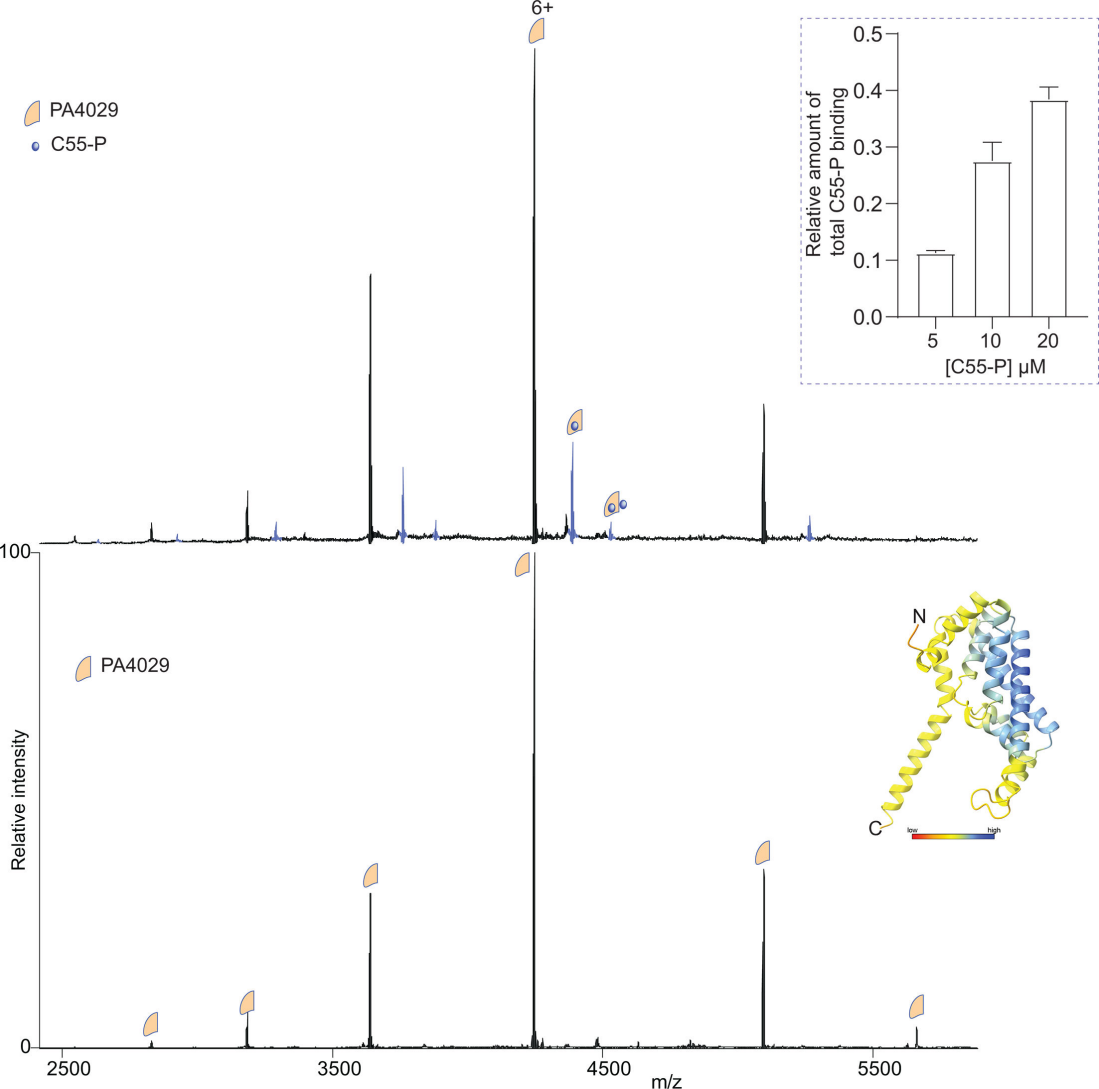
Binding of C55-P to PA4029. Native mass spectra of purified PA4029 (5 µM) with (top spectrum) and without (bottom spectrum) C55-P (5 µM). The data reveal adduct peaks corresponding to C55-P binding to PA4029 (blue series), with their intensity increasing as the C55-P concentration rises. Quantification of this data is displayed in the inset. Data are expressed as mean ± standard deviation (SD) of three independent replicates. Ribbon diagram is the model structure of PA4029 predicted by AlphaFold, colored by pLDDT (orange, 0–50; yellow, 50–70; cyan, 70–90; and blue, 90–100).

To determine whether C55-P binds to PA4029, we conducted binding experiments by incubating 5 µM PA4029 with increasing concentrations of C55-P (0–20 µM) and recording native MS data. Mass spectra revealed peaks for PA4029 in both its apo form and C55-P-bound form (Fig. 3). We observed that C55-P binds both monomeric and dimeric PA4029 (Fig. S4), with a higher affinity for dimeric PA4029, consistent with previous findings on UptA (41). The apparently lower level of C55-P binding to the monomer is likely due, at least partly, to the loss of bound lipid during the collisional activation needed to dissociate the detergent micelles. Overall, the dimeric data align with the trends seen in the monomer. Next, we deconvoluted the spectra to determine the mean relative intensities of C55-P-bound and unbound forms of PA4029 at all concentration points. The data showed that the intensity of the PA4029:C55-P complex increases as a function of C55-P concentration (Fig. 3). These results suggest a concentration-dependent interaction, further confirming that PA4029 binds to C55-P.

### Amphomycin disrupts the PA4029:C55-P complex

**Fig 4:**
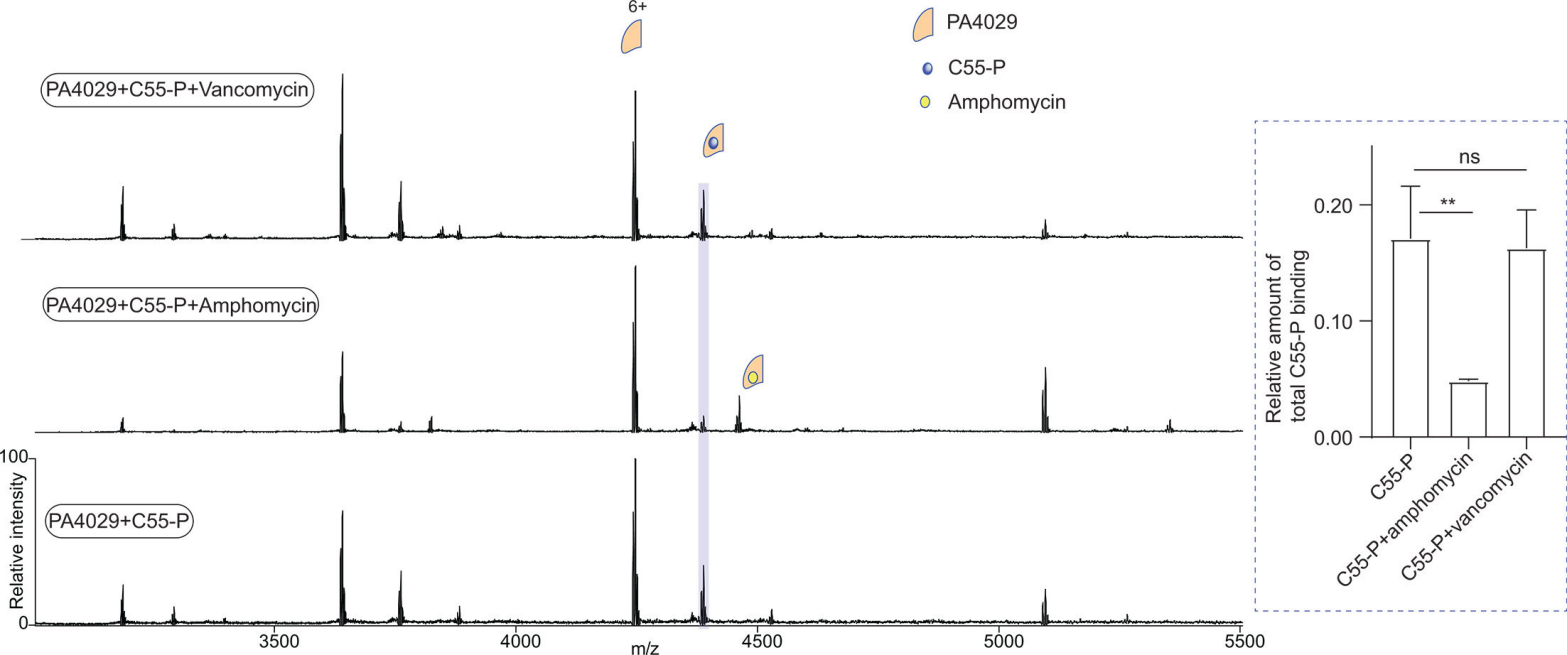
Amphomycin disrupts PA4029:C55-P interaction. Spectra of PA4029 with C55-P (bottom spectrum), C55-P and amphomycin (middle spectrum), and C55-P and vancomycin (top spectrum). The bar charts (inset) show the quantification of the amount of C55-P binding and suggest that the amount of PA4029:C55-P binding is significantly reduced in the presence of amphomycin. In contrast, the difference with vancomycin is not significant. Data are expressed as mean ± standard deviation (SD) of three independent replicates. Statistical significance was assessed using multiple *t*-tests, two-sided and unpaired (**P = 0.0017, ns = not significant).

To further confirm whether the interaction observed above is specific, we have focused on lipopeptide antibiotics that target cell wall synthesis. A recent study on *B. subtilis* UptA established a methodology to assess the effects of lipopeptide antibiotics on C55-P binding to DedA proteins and found that C55-P targeting antibiotics, such as amphomycin, bind to UptA and induce dissociation of the UptA:C55-P complex (41). To test whether amphomycin has a similar effect on the PA4029:C55-P complex, we performed binding studies with amphomycin. As a control, we included vancomycin, a glycopeptide antibiotic that targets lipid II but not C55-P. We recorded spectra for solutions containing 5 µM PA4029 and 10 µM C55-P, then equilibrated with 10 µM of each antibacterial compound. In the presence of amphomycin, the mass spectrum showed distinct peaks corresponding to amphomycin-PA4029 binding and a significant reduction in the peak intensity of the PA4029:C55-P complex (Fig. 4). In contrast, vancomycin had no appreciable effect on the PA4029:C55-P complex. These results indicate that amphomycin destabilizes the ternary complex, most likely by sequestering C55-P or impairing binding through competitive binding effects or conformational effects.

### PA4029 preferentially binds C55-P over phospholipids

**Fig 5:**
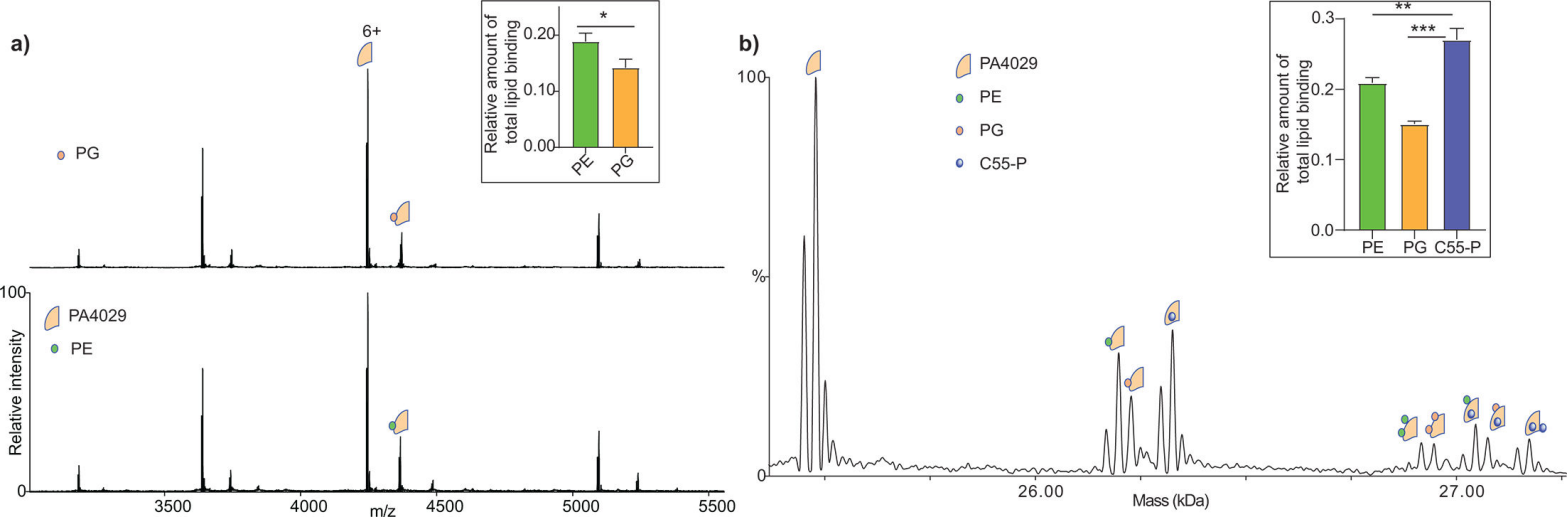
Phospholipid binding to PA4029 and its effect on C55-P binding. (**a**) Spectra of PA4029 in the presence of equal quantities of POPE and POPG. The relative intensity of lipid binding is higher in the case of PE than PG (insert). Data are expressed as mean ± standard deviation (SD) of three independent replicates. Statistical significance was assessed using multiple *t*-tests, two-sided and unpaired (*P = 0.02). (**b**) Deconvoluted spectrum of PA4029 with equal quantities of C55-P, POPE, and POPG. The bar charts (in inset) show the quantification of the amount of lipid binding and suggest that the amount of PA4029:C55-P binding is significantly higher than PA4029:POPE and PA4029:POPG. Additionally, ternary complexes are observed. Data are expressed as mean ± standard deviation (SD) of three independent replicates. Statistical significance was assessed using multiple *t*-tests, two-sided and unpaired (**P = 0.0041 and ***P = 0.0002).

Recent studies have highlighted the role of some DedA family transporters in phospholipid translocation (23). To determine whether PA4029 recognizes phospholipids and to evaluate their potential role in influencing C55-P interaction, we examined the binding of PA4029 to phosphatidylglycerol (PG) and PE. We obtained mass spectra of solutions with 5 µM PA4029 incubated with 10 µM PE (16:0–18:1) and 10 µM PG (16:0–18:1). In both cases, the spectra displayed peaks corresponding to PA4029 in both apo and lipid-bound forms (Fig. 5a and Fig. S5). We detected the formation of 1:1 and 1:2 protein-lipid complexes, with the mean relative intensity of the PA4029:PE complex exceeding that of the PA4029:PG complex (Fig. 5a), indicating that PA4029 interacts more favorably with PE than PG.

We also examined the relative affinity of C55-P binding compared to PE and PG (Fig. S5 and Fig. 5b). To achieve this, we recorded the spectra of 5 µM PA4029 in the presence of 10 µM C55-P, 10 µM PE, and 10 µM PG. We observed the formation of ternary complexes, indicating that the binding sites for C55-P and phospholipids are distinct. More importantly, we noted that the intensity of the PA4029:C55-P complex was significantly higher than that of the phospholipid-bound species (Fig. 5b). This supports the hypothesis that PA4029 binds more strongly to C55-P than to phospholipids, further suggesting its likely physiological role in recognizing and potentially recycling C55-P.

### Distribution of putative *Pseudomonas* undecaprenyl phosphate flippases

**Fig 6:**
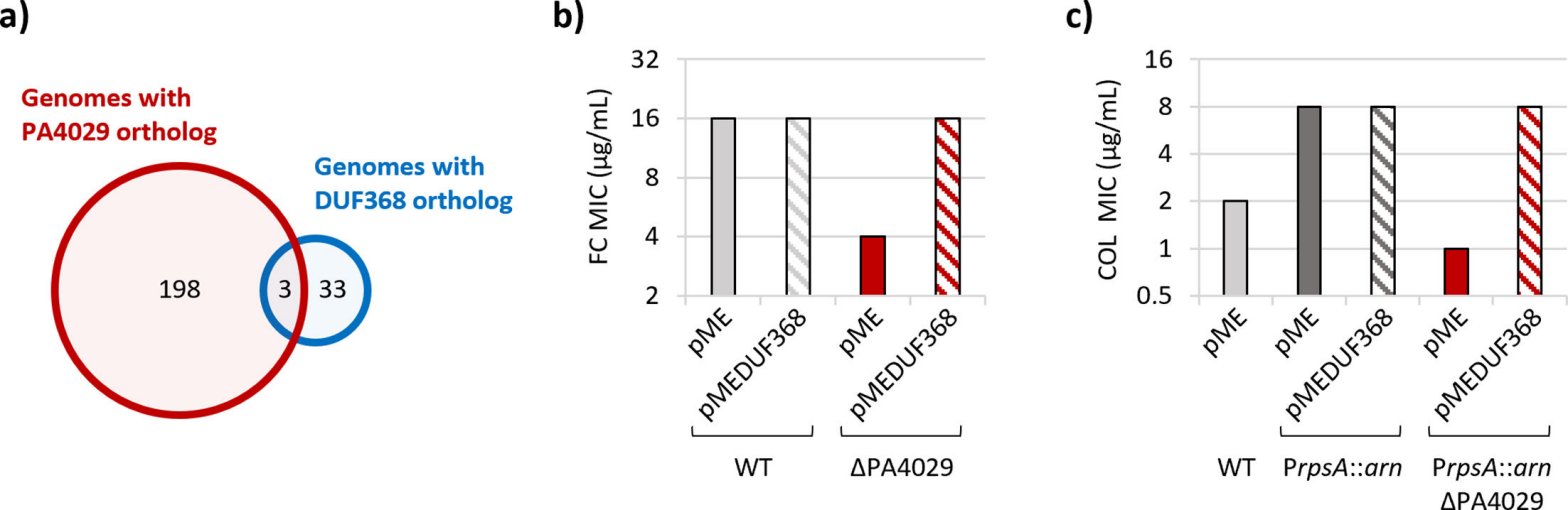
Distribution of PA4029 orthologs or DUF368 proteins in *Pseudomonas*. (**a**) Venn diagram showing the distribution of orthologs of PA4029 or DUF368 domain-containing protein across 236 genomes from different *Pseudomonas* species and strains. Note that two strains (*P. stutzeri* SLG510A3-8 and *Pseudomonas resinovorans* DSM 21078) apparently do not carry any PA4029 or DUF368 ortholog (Table S2). (**b**) Fosmidomycin (FC) MIC of *P. aeruginosa* PAO1 (WT) and the ΔPA4029 mutant carrying the empty plasmid pME6032 (pME) or its derivative for ectopic expression of the DUF368 domain-containing protein CXK92_RS12370 of *P. stutzeri* KC (pMEDUF368). (**c**) Colistin (COL) MIC of the WT, the recombinant strain P*rpsA::arn,* and its ΔPA4029 mutant carrying the empty plasmid pME or its derivative pMEDUF368. Values in panels b and c are the mode of at least three independent experiments.

Our *in vivo* results pointed out PA4029 as the primary DedA protein responsible for C55-P flip-back in *P. aeruginosa*, and *in vitro* data confirmed the capability of PA4029 to bind C55-P. However, while this protein is conserved in all *P. aeruginosa* strains analyzed in this study (Table 1), the search for orthologous genes in other *Pseudomonas* species revealed some species that lack PA4029 orthologs (Table 1; Table S2). In *V. cholerae*, *B. subtilis,* and *S. aureus*, proteins containing the DUF368 domain were also found to be involved in C55-P transport across the CM (18, 19). While DUF368 proteins were not identified in *P. aeruginosa* (Table S1; https://pseudomonas.com/), BLAST analysis revealed a homolog of the *V. cholerae* and *S. aureus* DUF368 proteins (VCA0040 and SAOUHSC_00846, respectively) in *Pseudomonas stutzeri* ATCC 17588 (Table S3), which was used as an example of a *Pseudomonas* strain lacking a PA4029 ortholog (Table S2). Interestingly, orthologs of the *P. stutzeri* ATCC 17588 DUF368 protein (PSTAB_1476) were identified in 33 out of 35 of the *Pseudomonas* strains without PA4029 orthologs, but only in 3 out of 201 strains carrying PA4029 orthologs (Table S2 and Fig. 6a), indicating that PA4029 orthologs and DUF368 proteins are mutually exclusive in *Pseudomonas*. This pattern suggests that the two proteins may serve a similar function, making the presence of both in the same strain redundant or unnecessary. To verify whether the DUF368 protein identified in some *Pseudomonas* species is involved in C55-P recycling, we ectopically expressed the ortholog of *P. stutzeri* KC (ATCC 55595) in *P. aeruginosa* cells lacking PA4029 and verified whether it can rescue the defects associated with impaired C55-P recycling. Notably, expression of the DUF368 protein restored both fosmidomycin resistance in cells lacking PA4029 (Fig. 6b) and the ability to acquire colistin resistance through lipid A aminoarabinosylation in the PAO1 P*rpsA::arn* ∆PA4029 recombinant strain (Fig. 6c), at levels identical to those obtained upon ectopic expression of PA4029 (Fig. 2e).

## DISCUSSION

Our study explores the role of DedA-family proteins in *P. aeruginosa*. Using bioinformatics, we screened for DedA family and DUF368 domain family members and characterized them functionally. We discovered that individual deletions of each DedA family member did not compromise cell growth and cell envelope integrity but that PA4029 is probably the major protein involved in C55-P recycling. Deleting PA4029 made cells more sensitive to fosmidomycin and made them less likely to acquire mutations conferring colistin resistance. These phenotypes are consistent with defects in C55-P recycling and suggest that PA4029 plays a role in this process. Notably, while this work was under revision, a manuscript was published reporting that PA4029 deletion causes accumulation of peptidoglycan precursors in the cytoplasm (42), consistent with the proposed role of PA4029 in C55-P recycling.

Native MS confirms that PA4029 binds C55-P directly, in a concentration-dependent manner. The complex is disrupted by the C55-P targeting antibiotic amphomycin but not by vancomycin. This is consistent with recent biochemical work with *B. subtilis* UptA (41). We also tested whether PA4029 binds other phospholipids (PE or PG). Although the protein formed complexes with both, C55-P binding was clearly favored in mixed-lipid assays, supporting the idea that PA4029 is not a general phospholipid transporter, but instead has a distinct affinity for C55-P. Unlike some other DedA proteins that may act as lipid scramblases or contribute to membrane homeostasis, PA4029 appears specific for C55-P.

**Fig 7:**
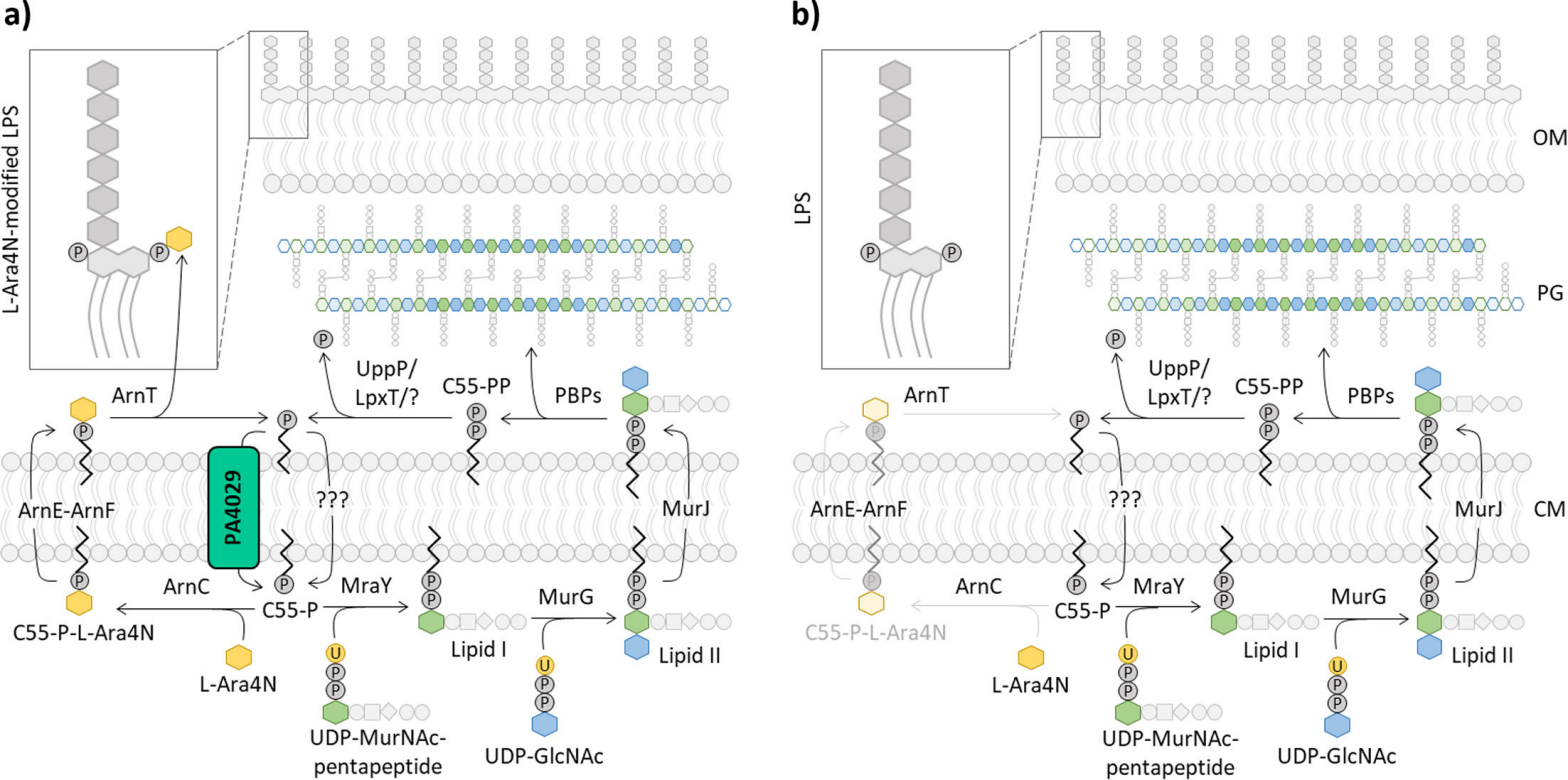
Schematic of PA4029 function*.* (**a**) PA4029 ensures proper recycling of C55-P for essential processes, such as cell wall biogenesis, and non-essential processes, such as lipid A aminoarabinosylation, mediated by Arn proteins. (**b**) PA4029 deficiency disrupts C55-P recycling, likely reducing the pool of C55-P available on the cytoplasmic side of the CM, which is mainly directed toward peptidoglycan biosynthesis to support growth, at the expense of lipid A aminoarabinosylation, thus impairing colistin resistance acquisition. GlcNAc, N-acetylglucosamine; MurNAc, N-acetylmuramic acid; LPS, lipopolysaccharide; PG, peptidoglycan; PBPs, penicillin binding proteins; OM, outer membrane.

In *P. aeruginosa*, colistin resistance is usually linked to the addition of L-Ara4N to lipid A, a process that requires C55-P as a lipid carrier for precursor transport. When C55-P recycling is disrupted, the absence of PA4029 likely reduces precursor flux, and the supply of lipid-linked intermediates becomes limited, which likely prevents L-Ara4N lipid A modification in favor of essential processes requiring C55-P, such as cell wall biogenesis (Fig. 7). This would explain why ΔPA4029 mutants show impaired emergence of colistin-resistant mutants and why PA4029 loss reverses colistin resistance and inhibits lipid A aminoarabinosylation under constitutive *arn* gene expression. Notably, defects in lipid A aminoarabinosylation were observed in *Burkholderia thailandensis* and *Klebsiella pneumoniae* mutants lacking specific DedA family proteins (26, 28), providing support to this hypothesis.

PA4011, another DedA homolog harboring a PAP2-like domain, displays a partial phenotype, as its absence reduces colistin resistance emergence but does not affect colistin MIC under constitutive *arn* expression. While functional overlap is possible, the inability of ectopically expressed PA4011 to compensate for PA4029 absence, along with the fact that PA4011 deletion does not worsen the defects caused by PA4029 deficiency, strongly suggests PA4011 plays a distinct or accessory role, perhaps in phosphoregulation or membrane lipid remodeling (43).

Interestingly, most *Pseudomonas* species lacking PA4029 possess a DUF368-family protein, and ectopic expression of one such DUF368 homolog from *P. stutzeri* fully rescues defects in fosmidomycin and colistin resistance in PA4029-deficient *P. aeruginosa* strains. This mutual distribution and functional complementation support the proposal that DedA and DUF368 proteins provide alternative solutions for C55-P flipping in the *Pseudomonas* genus. Future work should focus on direct reconstitution of flipping activity *in vitro*, high-resolution structural studies of PA4029:C55-P binding, and probing how DUF368 proteins fulfill this role in other *Pseudomonas* species.

## MATERIALS AND METHODS

### Bacterial strains, plasmids, and growth media

Strains and plasmids used in this work are listed in Tables S4 and S5, respectively. Bacteria were routinely cultured in Lysogeny broth, Lennox formulation (LB) for genetic manipulation, while growth and antibiotic susceptibility assays were performed in Mueller-Hinton broth (MH) and/or cation-adjusted MH (CAMH), as indicated.

### Generation of plasmids and mutants

To obtain the constructs for the generation of *P. aeruginosa* deletion mutants, two DNA fragments of approximately 500 bp encompassing the upstream and downstream regions of each gene of interest were amplified by PCR, directionally cloned into pBluescript II (pBS), and verified by DNA sequencing. Primers and restriction enzymes used for PCR and cloning or DNA sequencing are listed in Table S6. Then, the DNA fragment encompassing the upstream and downstream regions of each gene was excised from pBS and sub-cloned into the *sacB*-based suicide vector pDM4 (44). The resulting pDM4 derivatives (Table S5) were transferred into *P. aeruginosa* by conjugation, and the transconjugants were selected on plates containing 15 μg/mL nalidixic acid and 375 μg/mL chloramphenicol. Deletion mutations were obtained by recombination and sucrose-based selection as previously described (45), identified by PCR with the primer pairs UP_FW and DOWN_RV of each gene (Table S6), and confirmed by DNA sequencing.

The complementing plasmids pMEPA4011 and pMEPA4029 (Table S4) were generated by individually cloning the PCR-amplified coding sequence of PA4011 or PA4029 into the shuttle vector pME6032, downstream of the IPTG-inducible promoter (46). The plasmid pMEDUF368_Ps_ was generated by cloning the coding sequence of the gene CXK92_RS12370, encoding the DUF368 protein of *P. stutzeri* KC, into pME6032 downstream of the IPTG-inducible promoter. All inserts were verified by DNA sequencing. Primers and restriction enzymes used for PCR and cloning or DNA sequencing are listed in Table S6.

### Growth assay

Strains were precultured in MH, containing 50 μg/mL tetracycline in the case of strains carrying pME6032 or its derivatives, until late exponential phase and then refreshed 1:1,000 in fresh MH medium, supplemented with 30 μM IPTG for strains carrying pME6032 or its derivatives. Cultures were aliquoted in flat 96-well microtiter plates (200 µL in each well) and growth was measured as the optical density at 600 nm (OD_600_) of the bacterial cultures every 30 min in a Tecan Spark 10M microtiter plate reader.

### Membrane permeability assay

Exponential phase cells cultured in MH were harvested by centrifugation and resuspended in 5 mM HEPES (pH 7.2) at OD_600_ = 3. Equal volumes (150 μL) of bacterial suspensions and HEPES solution containing or not PI (40 μg/mL) were mixed, and 100 μL of each sample was aliquoted on a black flat-bottom 96-well plate. OD_600_ and fluorescence were measured in a Tecan Spark 10M microtiter plate reader (excitation at 580 nm and emission at 620 nm) after 2 min at room temperature, subtracted from the background values of samples without PI, and normalized to the OD_600_ of the cell suspension (47).

### Detergent sensitivity assay

Sensitivity to the lytic effect of SDS was assessed by determining the turbidity (OD_600_) of bacterial cell suspensions in saline after 5-min incubation at room temperature in the presence of increasing concentrations of SDS (0%–5%, wt/vol) (33).

### Antibiotic sensitivity assay

The resistance profile of bacterial strains to several antibiotics was assessed by the disc diffusion test. Strains were cultured in MH until late exponential phase, centrifuged, resuspended at a theoretical OD_600_ = 0.08 in sterile saline solution, and swabbed onto MH agar plates. Disks containing rifampicin (30 µg) (Liofilchem), ciprofloxacin (5 µg), colistin (10 µg), aztreonam (30 µg), streptomycin (10 µg), gentamycin (10 µg), imipenem (10 µg), novobiocin (30 µg), tobramycin (10 µg), and erythromycin (15 µg) (Becton Dickinson) were placed on the agar surface before incubation of plates. The diameters of the growth inhibition halos were measured after 24 h of incubation at 37°C.

### MIC assays

The MIC of antibiotics was determined through the broth microdilution method. *P. aeruginosa* strains were precultured in MH for fosmidomycin MIC assay or CAMH for colistin MIC assay and then refreshed in the same medium at ca. 5 × 10^5^ cells/mL in the presence of increasing concentrations of each antibiotic. MIC was defined as the lowest antibiotic concentration for which no visible growth was observed after 24 h of incubation at 37°C under static conditions.

### Frequency of colistin-resistant mutants

To evaluate the frequency of emergence of colistin-resistant spontaneous mutants, strains were cultured in MH at 37°C until the late exponential phase, harvested by centrifugation, and resuspended in sterile saline solution at a theoretical OD_600_ = 1. Serial 10-fold dilutions in saline were prepared and spotted onto LB agar plates to measure the number of colony-forming units (CFUs). Mutants were selected by plating 200 µL aliquots of the OD_600_ = 1 suspensions onto MH agar plates supplemented with 10 µg/mL colistin (and 0.031 mM IPTG for strains carrying pME6032 or its derivatives) and incubated at 37°C for 72 h. Frequency of colistin-resistant spontaneous mutants was calculated as the ratio between the number of colonies that appeared on MH agar plates supplemented with colistin and the total number of CFUs plated.

### Protein expression and purification

A codon-optimized version of *P. aeruginosa* PA4029 (Integrated DNA Technologies) was inserted into NdeI and NheI restriction sites (by In-Fusion HD cloning kit, Takara BioTM) of a modified pET15b vector that adds a TEV-GFP-6×His tag at the end of the protein (41) and verified by sequencing. The resulting plasmid was inserted into *E. coli* C43 (DE3) cells (Lucigen) by transformation. For large-scale protein expression, 10 mL of overnight culture was transferred into 1 L of LB containing 100 µg/mL ampicillin, and cells were allowed to grow at 37°C until the OD_600_ reached 0.6–0.8, after which the temperature was lowered to 18°C, and the expression was induced with 0.5 mM IPTG for 16 h. Cells were harvested by centrifugation, pellets were resuspended in resuspension buffer (20 mM Tris-HCl, pH 8.0, 200 mM NaCl, 10% glycerol) and lysed immediately or N_2_ flash-frozen and stored at −80°C until required.

Cells were thawed on ice and resuspended in a buffer (20 mM Tris-HCl, pH 8.0, 200 mM NaCl, 10% glycerol) containing lysozyme and protease-inhibitor cocktail tablets and then lysed using sonication (total processing time = 10 min, pulse-on:pulse-off = 3:6, amplitude = 65 with a Mili-tip). Non-lysed cells and debris were pelleted by centrifugation at 20,000 × *g* for 20 min at 4°C, and the clarified lysate was retained. The membrane fraction (supernatant) was then pelleted by ultracentrifugation at 160,000 × *g* for 1 h at 4°C and resuspended in a buffer containing 20 mM Tris-HCl (pH 8.0), 200 mM NaCl, 20% glycerol. Membrane aliquots were solubilized immediately or flash-frozen and stored at −80°C. The membrane was thawed and made up to a volume of 25 mL with resuspension buffer and solubilized by incubation with 2% DDM for 1 h at 4°C. The solubilized fraction was centrifuged at 20,000 × *g* for 20 min at 4°C to remove non-solubilized aggregates. The supernatant containing the membrane proteins was loaded onto a 5-mL HisTrap nickel nitriloacetic acid (Ni-NTA) agarose resin column pre-equilibrated in a buffer (20 mM Tris-HCl, pH 8.0, 200 mM NaCl, 10% glycerol, 0.03% DDM, 20 mM imidazole). Ni-NTA beads were taken out into a Falcon tube and placed on a rotor for 1 h at 4°C. After binding of the His-tagged protein, the mixture was loaded back to the column and washed with 100 mL buffer (20 mM Tris-HCl, pH 8.0, 200 mM NaCl, 10% glycerol, 0.03% DDM, 20 mM imidazole), and then with 50 mL of another buffer (20 mM Tris-HCl, pH 8.0, 200 mM NaCl, 10% glycerol, 0.03% DDM, 80 mM imidazole). PA4029-GFP was eluted from the column with elution buffer (20 mM Tris-HCl, pH 8.0, 200 mM NaCl, 10% glycerol, 0.03% DDM, 200 mM imidazole). To remove imidazole and to cleave the GFP fusion, the eluted protein was mixed with TEV proteases and dialyzed against dialysis buffer (20 mM Tris-HCl, pH 8.0, 200 mM NaCl, 10% glycerol, 0.03% DDM) at 4°C for 20 h. The digested mixture was subsequently incubated with 3 mL of Ni-NTA agarose resin to remove His-tagged GFP and TEV protease. Tag-free PA4029 was collected as the flowthrough, concentrated using a SartoriusTM Vivaspin centrifugal concentrator with a nominal molecular weight cut-off of 50 kDa, and finally purified by size-exclusion chromatography (SEC) using a 24-mL Superdex 200 Increase column. The SEC buffer was 20 mM Tris-HCl, pH 8.0, 200 Mm NaCl, 0.03% DDM. After SEC purification, PA4029 was concentrated to 200 μM, aliquoted (50 μM each), flash-frozen in N_2_, and stored at −80°C until required. Concentration was measured using a Nanodrop UV spectrophotometer at 280 nm.

### Native MS

Protein was buffer exchanged into 200 mM ammonium acetate (pH 7.0) and 0.05% LDAO using BioSpin-6 (BIORAD) columns. About 3 μL protein sample was introduced directly into the mass spectrometer, Q-Exactive UHMR (Thermo Fisher Scientific, Bremen, Germany), using a gold-coated borosilicate capillary (prepared in-house, originally from Harvard Apparatus). The instrument settings were as follows: capillary voltage 1.2 kV, S-lens RF 100%, quadrupole selection from 1,000 to 20,000 m/z range, collisional activation in the HCD cell 120 V, trapping gas pressure setting 7.5, temperature 200°C, and resolution of the instrument was set to 12,500. The noise level was set at 3 rather than the default value of 4.64. No in-source dissociation was applied. Data were exported for processing using the Qual browser of Xcalibur 4.2 (Thermo Scientific), and spectral deconvolution was performed using UniDec software packages. Relative binding affinities were obtained from deconvoluted spectra by dividing the intensity of ligand-bound protein peaks by the sum of the intensities of ligand-bound and ligand-free protein peaks.

### Lipid A analysis by matrix-assisted laser desorption/ionization–time of flight

Matrix-assisted laser desorption/ionization–time of flight (MALDI-TOF) MS spectra were acquired in resolution mode and negative ion polarity on a Waters SYNAPT XS mass spectrometer (Waters, Manchester, UK) equipped with an 8 kDa quadrupole and a MALDI source. Lipid A was extracted as previously described (48) from dried bacterial pellets obtained from early stationary phase bacterial cultures in MH, and then dissolved in chloroform/methanol (50:50, vol:vol), while the matrix solution was 2,4,6-trihydroxyacetophenone (Sigma Aldrich) dissolved in methanol/0.1% trifluoroacetic acid/acetonitrile (7:2:1, vol:vol:vol) at concentration of 75 mg/mL. A volume of 0.5 μL of the sample and 0.5 μL of the matrix solution were applied to the MALDI plate and allowed to air dry at room temperature. Spots were randomly but evenly sampled, and experiments were executed in triplicate.

### Bioinformatic analyses

TMHs were predicted using DeepTMHMM (https://dtu.biolib.com/DeepTMHMM) (49). Predicted 3D structures were retrieved from the AlphaFold database (https://alphafold.ebi.ac.uk/) (50). Homologous proteins were identified using BLASTP searches in the *Pseudomonas* Genome Database (https://pseudomonas.com/) and the amino acid sequence of proteins of interest as the query. Orthologs within the *Pseudomonas* genus were identified using the Ortholog Group Members function of the *Pseudomonas* Genome Database (51).

### Statistics

Statistical analysis was performed with the software GraphPad Instat, using the one-way analysis of variance (ANOVA), followed by the Kruskal-Wallis multiple comparisons test.

## Data Availability

The authors confirm that the data supporting the findings of this study are available in the article and its supplemental material.

## SUPPLEMENTAL MATERIAL

Supplemental Material mbio.02408-25-s0001.pdf Tables S1 to S6 and Fig. S1 to S5.

ASM does not own the copyrights to Supplemental Material that may be linked to, or accessed through, an article. The authors have granted ASM a non-exclusive, world-wide license to publish the Supplemental Material files. Please contact the corresponding author directly for reuse.
