# Supplementary material for: The Pseudomonas aeruginosa DedA protein PA4029 is an undecaprenyl phosphate flippase important for polymyxin resistance
Source: mBio. 2026 Jan 12;17(2):e02408-25. doi: 10.1128/mbio.02408-25 (PMC12893004; doi:10.1128/mbio.02408-25)
Supplement: Supplemental Material — Tables S1 to S6 and Fig. S1 to S5. [file mbio.02408-25-s0001.pdf]

The *Pseudomonas aeruginosa* DedA protein PA4029 is an undecaprenyl phosphate flippase important for polymyxin resistance

Davide Sposato, Yi Wang, Xinye Zhang, Ludovica Rossi, Stefania De Chiara, Flaviana Di Lorenzo, Giordano Rampioni, Livia Leoni, Paolo Visca, Jani Bolla, Francesco Imperi

SUPPLEMENTARY MATERIAL

Table S1. DedA proteins identified in *P. aeruginosa* PAO1 by homology search.

Table S2. *Pseudomonas* strains with orthologs of *P. aeruginosa* PAO1 DedA proteins and *P. stutzeri* KC DUF368 protein CXK92_RS12370.

Table S3. Homolog of previously characterized DUF368 proteins identified in *P. stutzeri* ATCC 17588 by homology search.

Table S4. Bacterial strains used in this study.

Table S5. Plasmids used in this study.

Table S6. Primers used in this study.

Figure S1. Predicted three-dimensional structure and transmembrane helices of the six *P. aeruginosa* DedA-like proteins.

Figure S2. Lipid A analysis by MALDI-TOF MS.

Figure S3. Native mass spectrum of PA4029.

Figure S4. C55-P binding to PA4029.

Figure S5. C55-P and phospholipids binding to PA4029.

Table S1. Homologs of previously characterized DedA proteins identified in *P. aeruginosa* PAO1 by homology search.[a]

Query	Function (Reference)	Protein family	*P. aeruginosa* PAO1 homolog(s) (PA number)	Product	Alignment length (aa)	% Identity	E-value
Escherichia coli YabI	Unknown	DedA	PA4011	hypothetical protein	152	38.816	1.76E-30
			PA4029	conserved hypothetical protein	155	25.161	3.74E-08
Escherichia coli YohD	Unknown	DedA	PA5244	conserved hypothetical protein	159	37.736	4.09E-31
			PA4011	hypothetical protein	141	29.078	2.23E-06
			PA4029	conserved hypothetical protein	162	24.074	2.08E-06
Escherichia coli DedA	Unknown	DedA	PA4029	conserved hypothetical protein	204	59.314	0
			PA4011	hypothetical protein	192	25.521	1.66E-12
			PA5244	conserved hypothetical protein	148	26.351	2.24E-07
			PA1209	hypothetical protein	131	25.191	2.57E-06
Escherichia coli YqjA	Unknown	DedA	PA4029	conserved hypothetical protein	177	35.593	3.71E-26
			PA4011	hypothetical protein	160	31.875	8.33E-14
			PA5244	conserved hypothetical protein	150	28.000	7.89E-07
Escherichia coli YghB	Unknown	DedA	PA4029	conserved hypothetical protein	157	34.395	3.35E-23
			PA5244	conserved hypothetical protein	150	32.667	8.73E-09
			PA4011	hypothetical protein	160	28.750	2.56E-07
Escherichia coli YdjX[b]	Unknown	DedA	PA4029	conserved hypothetical protein	120	29.167	0.829999983
			PA3725	single-stranded-DNA-specific exonuclease RecJ	58	25.862	1.200000048
			PA5413	low specificity l-threonine aldolase	32	43.750	1.799999952
Escherichia coli YdjZ[b]	Unknown	DedA	PA4029	conserved hypothetical protein	113	26.549	0.000114
			PA1301	HxuR	105	27.619	0.779999971
			PA3220	probable transcriptional regulator	98	24.490	0.769999981
			PA0537	conserved hypothetical protein	27	33.333	1.5
Escherichia coli YqaA	Unknown	DedA	PA2752	conserved hypothetical protein	131	45.802	5.77E-21
Vibrio cholerae YghB (VCA0534)	Undecaprenyl phosphate translocase (Sit *et al.* 2023,10.1038/s41586-022-05569-1)	DedA	PA4029	conserved hypothetical protein	155	30.968	2.61E-11
			PA5244	conserved hypothetical protein	149	30.201	8.46E-08
			PA4011	hypothetical protein	204	26.471	1.4E-06
Bacillus subtilis UptA (YngC)	Undecaprenyl phosphate translocase (Roney and Rudner 2023, 10.1038/s41586-022-05587-z)	DedA	PA4029	conserved hypothetical protein	93	31.183	3.3E-08
			PA4011	hypothetical protein	130	25.385	9.93E-08
Bacillus subtilis PetA (YbfM)	Phosphatidylethanolamine translocase (flippase) (Roney and Rudner 2023, 10.1073/pnas.2301979120)	DedA	PA5244	conserved hypothetical protein	146	25.342	6.25E-10
			PA4011	hypothetical protein	150	28	3.37E-08
			PA4029	conserved hypothetical protein	161	23.602	0.00000006
			PA1209	hypothetical protein	144	23.611	0.000015
Staphylococcus aureus SAOUHSC_02816	Undecaprenyl phosphate translocase (Sit *et al.* 2023,10.1038/s41586-022-05569-1)	DedA	PA4029	conserved hypothetical protein	166	26.506	5.52E-12
			PA5244	conserved hypothetical protein	148	23.649	8.31E-10
			PA4011	hypothetical protein	148	25.676	0.0000257
Pseudomonas aeruginosa PA1209	Unknown	DedA	PA4029	conserved hypothetical protein	163	28.834	1.15E-12
			PA5244	conserved hypothetical protein	170	24.706	1.24E-05
Pseudomonas aeruginosa PA2752[b]	Unknown	DedA	PA4029	conserved hypothetical protein	136	28.676	0.000836
			PA4011	hypothetical protein	85	31.765	0.129999995
			PA5517	conserved hypothetical protein	139	23.022	1.299999952
			PA0282	sulfate transport protein CysT	50	36.000	1.600000024
			PA5244	conserved hypothetical protein	138	25.362	1.899999976
Pseudomonas aeruginosa PA4011	Unknown	DedA	PA4029	conserved hypothetical protein	169	27.811	6.03E-13
			PA5244	conserved hypothetical protein	140	30.000	2.34E-05
Pseudomonas aeruginosa PA4029	Unknown	DedA	PA4011	hypothetical protein	169	27.811	1.53E-13
			PA5244	conserved hypothetical protein	148	27.703	1.77E-08
			PA1209	hypothetical protein	145	27.586	8.66E-08
Pseudomonas aeruginosa PA5244	Unknown	DedA	PA4029	conserved hypothetical protein	173	28.324	1.55E-09
			PA4011	hypothetical protein	140	30.000	1.55E-05
Pseudomonas aeruginosa PA5517[b]	Unknown	DedA	PA5028	conserved hypothetical protein	96	21.875	1.700000048
			PA2752	conserved hypothetical protein	139	23.022	1.799999952
Vibrio cholerae VCA0040[b]	Undecaprenyl phosphate translocase (Sit *et al.* 2023,10.1038/s41586-022-05569-1)	DUF368	PA3781	probable transporter	115	26.087	0.939999998
Staphylococcus aureus SAOUHSC_00846[b]	Undecaprenyl phosphate translocase (Roney and Rudner 2023, 10.1038/s41586-022-05587-z)	DUF368	PA0108	cytochrome c oxidase, subunit III	42	47.619	0.97

[a] Unless otherwise stated, homologs were identified by BLASTP analysis with an E-value cutoff of 1×e-4 (default value for BLASTP analysis in the *Pseudomonas* Genome Database).

[b] Since BLASTP analysis with an E-value cutoff of 1×e-4 did not retrieve any homolog, very distant homologs were searched using an E-value cutoff of 2.

Table S2. ***Pseudomonas*** **strains with orthologs of** ***P. aeruginosa*** **PAO1 DedA proteins and** ***P. stutzeri*** **KC DUF368 protein CXK92_RS12370.**[a]

Strain	Ortholog of						
	P. aeruginosa DedA protein						*P. stutzeri* DUF368 protein
	PA1209	PA2752	PA4011	PA4029	PA5244	PA5517	CXK92_RS12370
Pseudomonas alcaliphila 34							
Pseudomonas alkylphenolia KL28							
Pseudomonas amygdali pv. *tabaci* ATCC 11528							
Pseudomonas balearica DSM6083 (=SP1402)							
Pseudomonas brassicacearum 51MFCVI2.1							
Pseudomonas brassicacearum DF41							
Pseudomonas brassicacearum PA1G7							
Pseudomonas brassicacearum PP1_210F							
Pseudomonas brassicacearum subsp. *brassicacearum* NFM421							
Pseudomonas caeni DSM 24390							
Pseudomonas chloritidismutans AW-1							
Pseudomonas chlororaphis EA105							
Pseudomonas chlororaphis HT66							
Pseudomonas chlororaphis O6							
Pseudomonas chlororaphis PA23							
Pseudomonas chlororaphis PCL1606							
Pseudomonas chlororaphis subsp. *aurantiaca* JD37							
Pseudomonas chlororaphis subsp. *aureofaciens* 30-84							
Pseudomonas chlororaphis subsp. *aureofaciens* NBRC 3521							
Pseudomonas chlororaphis UFB2 isolate Soil							
Pseudomonas chlororaphis YL-1							
Pseudomonas cichorii JBC1							
Pseudomonas cremoricolorata DSM 17059							
Pseudomonas cremoricolorata ND07							
Pseudomonas deceptionensis DSM 26521							
Pseudomonas denitrificans ATCC 13867							
Pseudomonas entomophila L48							
Pseudomonas fluorescens AU10414							
Pseudomonas fluorescens AU10973							
Pseudomonas fluorescens AU11114							
Pseudomonas fluorescens AU11122							
Pseudomonas fluorescens AU11136							
Pseudomonas fluorescens AU11164							
Pseudomonas fluorescens AU11235							
Pseudomonas fluorescens AU12597							
Pseudomonas fluorescens AU12644							
Pseudomonas fluorescens AU13852							
Pseudomonas fluorescens AU20219							
Pseudomonas fluorescens AU2390							
Pseudomonas fluorescens AU5633							
Pseudomonas fluorescens AU6026							
Pseudomonas fluorescens AU6308							
Pseudomonas fluorescens AU7350							
Pseudomonas fluorescens C1							
Pseudomonas fluorescens C2							
Pseudomonas fluorescens C3							
Pseudomonas fluorescens EGD-AQ6							
Pseudomonas fluorescens F113							
Pseudomonas fluorescens MEP34							
Pseudomonas fluorescens PA4C2							
Pseudomonas fluorescens PCL1751							
Pseudomonas fluorescens Pf0-1							
Pseudomonas fluorescens PICF7							
Pseudomonas fluorescens Q2-87							
Pseudomonas fluorescens R124							

Pseudomonas fluorescens SBW25							
Pseudomonas fluorescens SF39a							
Pseudomonas fluorescens SF4c							
Pseudomonas fluorescens SS101							
Pseudomonas fluorescens UK4							
Pseudomonas frederiksbergensis SI8							
Pseudomonas fulva 12-X							
Pseudomonas fulva NBRC 16636 = DSM 17004							
Pseudomonas helleri DSM 29165							
Pseudomonas kilonensis 1855-344							
Pseudomonas knackmussii B13							
Pseudomonas lini DSM 16768							
Pseudomonas lundensis DSM 6252							
Pseudomonas lutea DSM 17257							
Pseudomonas mandelii 36MFCvi1.1							
Pseudomonas mandelii JR-1							
Pseudomonas mediterranea CFBP 5447							
Pseudomonas mendocina EGD-AQ5							
Pseudomonas mendocina NBRC 14162							
Pseudomonas mendocina NK-01							
Pseudomonas monteilii NBRC 103158 = DSM 14164							
Pseudomonas monteilii SB3078							
Pseudomonas monteilii SB3101							
Pseudomonas moraviensis R28-S							
Pseudomonas mosseli i DSM 17497							
Pseudomonas mosselii SJ10							
Pseudomonas nitroreducens Aramco J							
Pseudomonas oleovorans MOIL14HWK12							
Pseudomonas oryzihabitans NBRC 102199							
Pseudomonas oryzihabitans RIT370							
Pseudomonas otitidis LNU-E-001							
Pseudomonas parafulva CRS01-1							
Pseudomonas parafulva NBRC 16636 = DSM 17004							
Pseudomonas parafulva YAB-1							
Pseudomonas pelagia 58							
Pseudomonas plecoglossicida NB2011							
Pseudomonas plecoglossicida NBRC 103162 = DSM 15088							
Pseudomonas plecoglossicida NyZ12							
Pseudomonas poae RE*1-1-14							
Pseudomonas protegens CHA0							
Pseudomonas protegens Pf-5							
Pseudomonas psychrotolerans L19							
Pseudomonas putida BIRD-1							
Pseudomonas putida DLL-E4							
Pseudomonas putida GB-1							
Pseudomonas putida H							
Pseudomonas putida H8234							
Pseudomonas putida HB3267							
Pseudomonas putida KG-4							
Pseudomonas putida LS46							
Pseudomonas putida NBRC 14164							
Pseudomonas putida PA14H7							
Pseudomonas putida PD1							
Pseudomonas putida S12							
Pseudomonas putida S13.1.2							
Pseudomonas putida S16							
Pseudomonas putida S610							
Pseudomonas putida YKD221							
Pseudomonas resinovorans DSM 21078							
Pseudomonas resinovorans NBRC 106553							
Pseudomonas rhizosphaerae DSM 16299							
Pseudomonas savastanoi pv. *phaseolicola* 1448A							
Pseudomonas simiae 2-36							

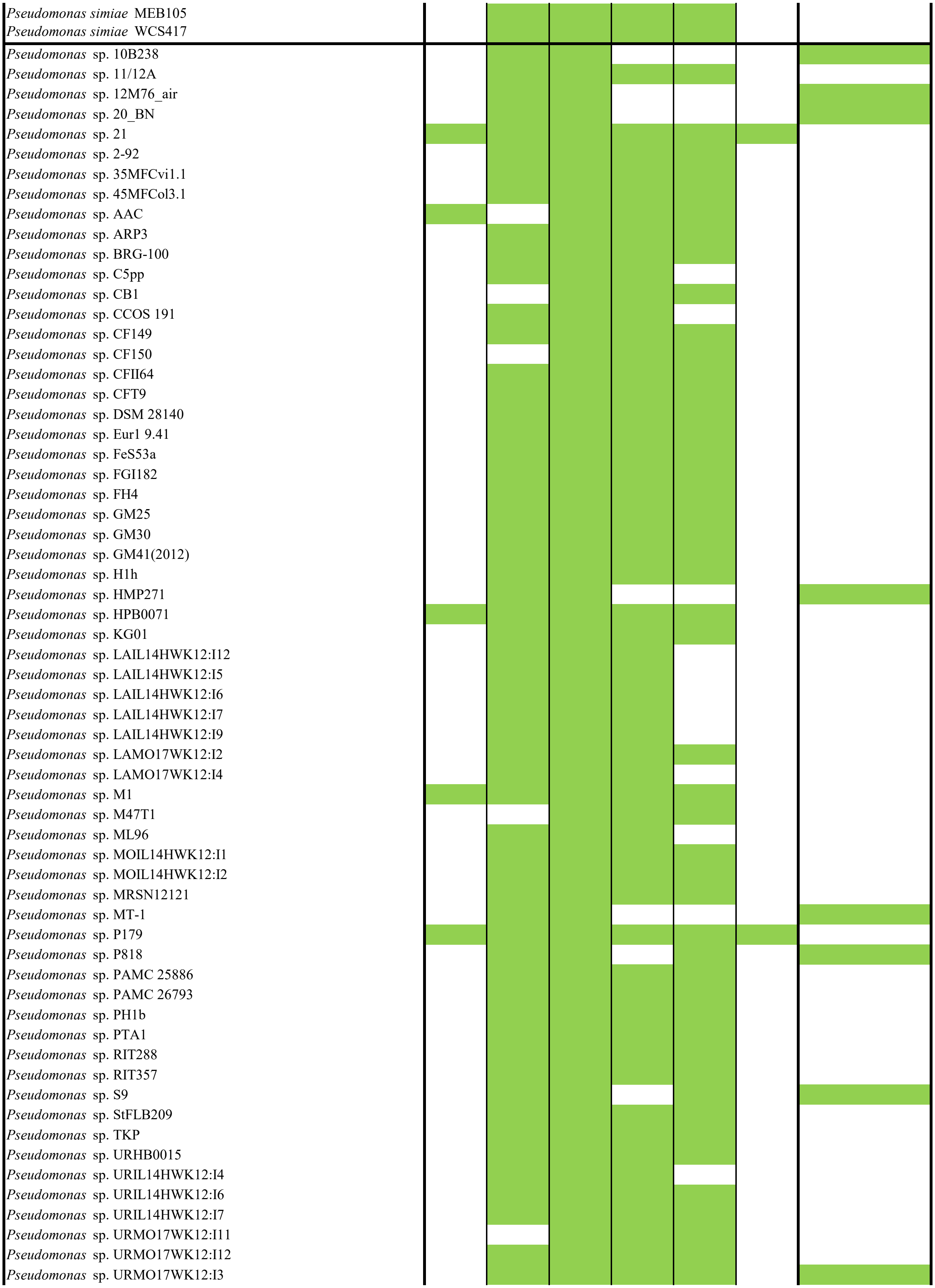

Pseudomonas simiae MEB105
Pseudomonas simiae WCS417
Pseudomonas sp. 10B238
Pseudomonas sp. 11/12A
Pseudomonas sp. 12M76_air
Pseudomonas sp. 20_BN
Pseudomonas sp. 21
Pseudomonas sp. 2-92
Pseudomonas sp. 35MFCvi1.1
Pseudomonas sp. 45MFCol3.1
Pseudomonas sp. AAC
Pseudomonas sp. ARP3
Pseudomonas sp. BRG-100
Pseudomonas sp. C5pp
Pseudomonas sp. CB1
Pseudomonas sp. CCOS 191
Pseudomonas sp. CF149
Pseudomonas sp. CF150
Pseudomonas sp. CFII64
Pseudomonas sp. CFT9
Pseudomonas sp. DSM 28140
Pseudomonas sp. Eur1 9.41
Pseudomonas sp. FeS53a
Pseudomonas sp. FGI182
Pseudomonas sp. FH4
Pseudomonas sp. GM25
Pseudomonas sp. GM30
Pseudomonas sp. GM41(2012)
Pseudomonas sp. H1h
Pseudomonas sp. HMP271
Pseudomonas sp. HPB0071
Pseudomonas sp. KG01
Pseudomonas sp. LAIL14HWK12:I12
Pseudomonas sp. LAIL14HWK12:I5
Pseudomonas sp. LAIL14HWK12:I6
Pseudomonas sp. LAIL14HWK12:I7
Pseudomonas sp. LAIL14HWK12:I9
Pseudomonas sp. LAMO17WK12:I2
Pseudomonas sp. LAMO17WK12:I4
Pseudomonas sp. M1
Pseudomonas sp. M47T1
Pseudomonas sp. ML96
Pseudomonas sp. MOIL14HWK12:I1
Pseudomonas sp. MOIL14HWK12:I2
Pseudomonas sp. MRSN12121
Pseudomonas sp. MT-1
Pseudomonas sp. P179
Pseudomonas sp. P818
Pseudomonas sp. PAMC 25886
Pseudomonas sp. PAMC 26793
Pseudomonas sp. PH1b
Pseudomonas sp. PTA1
Pseudomonas sp. RIT288
Pseudomonas sp. RIT357
Pseudomonas sp. S9
Pseudomonas sp. StFLB209
Pseudomonas sp. TKP
Pseudomonas sp. URHB0015
Pseudomonas sp. URIL14HWK12:I4
Pseudomonas sp. URIL14HWK12:I6
Pseudomonas sp. URIL14HWK12:I7
Pseudomonas sp. URMO17WK12:I11
Pseudomonas sp. URMO17WK12:I12
Pseudomonas sp. URMO17WK12:I3

Pseudomonas sp. URMO17WK12:I4							
Pseudomonas sp. URMO17WK12:I8							
Pseudomonas sp. UW4							
Pseudomonas sp. VLB120							
Pseudomonas sp. WCS358							
Pseudomonas sp. WCS374							
Pseudomonas stutzeri 19SMN4							
Pseudomonas stutzeri 28a24							
Pseudomonas stutzeri A1501							
Pseudomonas stutzeri ATCC 17588 = LMG 11199							
Pseudomonas stutzeri B1SMN1							
Pseudomonas stutzeri CCUG 29243							
Pseudomonas stutzeri DSM 10701							
Pseudomonas stutzeri DSM 4166							
Pseudomonas stutzeri KOS6							
Pseudomonas stutzeri MF28							
Pseudomonas stutzeri NF13							
Pseudomonas stutzeri NT0124							
Pseudomonas stutzeri NT0128							
Pseudomonas stutzeri RCH2							
Pseudomonas stutzeri SLG510A3-8							
Pseudomonas stutzeri T13							
Pseudomonas stutzeri TS44							
Pseudomonas synxantha BG33R							
Pseudomonas syringae B576							
Pseudomonas syringae BRIP39023							
Pseudomonas syringae CC1557							
Pseudomonas syringae DSM 10604							
Pseudomonas syringae pv. *actinidiae* ICMP 9617							
Pseudomonas syringae pv. *coryli* NCPPB 4273							
Pseudomonas syringae pv. *syringae* 41a							
Pseudomonas syringae pv. *syringae* B301D							
Pseudomonas syringae pv. *syringae* B301D-R							
Pseudomonas syringae pv. *syringae* B64							
Pseudomonas syringae pv. *syringae* B728a							
Pseudomonas syringae pv. *syringae* HS191							
Pseudomonas syringae pv. syringae SM							
Pseudomonas syringae pv. *tomato* DC3000							
Pseudomonas syringae pv. *tomato* NYS-T1							
Pseudomonas syringae UB0390							
Pseudomonas taeanensis MS-3							
Pseudomonas taetrolens DSM 21104							
Pseudomonas taiwanensis DSM 21245							
Pseudomonas thermotolerans DSM 14292							
Pseudomonas thermotolerans J53							
Pseudomonas trivialis IHBB745							
Pseudomonas tuomuerensis JCM 14085							
Pseudomonas umsongensis 20MFCvi1.1							
Pseudomonas umsongensis UNC430CL58Col							
Pseudomonas veronii 1YdBTEX2							
Pseudomonas veronii R4							
Pseudomonas viridiflava LMCA8							
Pseudomonas vranovensis DSM 16006							
Pseudomonas weihenstephanensis DSM 29166							

[a] Green and white cells indicate the presence or absence of an ortholog, respectively.

Table S3. Homolog of previously characterized DUF368 proteins identified in *P. stutzeri* ATCC 17588 by homology search.[a]

Query	Function (Reference)	Protein family	*P. stutzeri* ATCC 17588 homolog	Product	Alignment length (aa)	% Identity	E-value
Vibrio cholerae VCA0040	Undecaprenyl phosphate translocase (Sit *et al.* 2023,10.1038/s41586-022-05569-1)	DUF368	PSTAB_1476	hypothetical protein	302	56.954	0
Staphylococcus aureus SAOUHSC_00846	Undecaprenyl phosphate translocase (Roney and Rudner 2023, 10.1038/s41586-022-05587-z)	DUF368	PSTAB_1476	hypothetical protein	251	33.466	5.78E-26

[a] Homologs were identified by BLASTP analysis with an E-value cutoff of 1×e-4 (default value for BLASTP analysis in the *Pseudomonas* Genome Database).

Table S4. *P. aeruginosa* strains used in this study.

Strain	Genotype and/or relevant characteristics	Source/reference
E. coli		
S17.1λ*pir*	*thi pro hsdR hsdM*$^+$ *recA* RP4-2-Tc::Mu-Km::Tn7*λpir,* Gm^R	Simon *et al.*, 1983 [a]
DH5αF'	*recA1 endA1 hsdR17 supE44 thi-1 gyrA96 relA1* Δ(*lacZYA-argF*)U169[φ*80 dlacZ*Δ*M15*], Nal^R	Liss, 1987 [a]
C43(DE3)	BL21(DE3) derivative for overexpression of toxic and/or membrane proteins	Lucigen/Miroux and Walker, 1996 [a]
P. aeruginosa		
PAO1 (ATCC15692)	Reference isolate, wild type	American Type Culture Collection
ΔPA1209	PAO1 derivative with an in-frame deletion of the PA1209 coding sequence	This work
ΔPA2752	PAO1 derivative with an in-frame deletion of the PA2752 coding sequence	This work
ΔPA4011	PAO1 derivative with an in-frame deletion of the PA4011 coding sequence	This work
ΔPA4029	PAO1 derivative with an in-frame deletion of the PA4029 coding sequence	This work
ΔPA5244	PAO1 derivative with an in-frame deletion of the PA5244 coding sequence	This work
ΔPA5517	PAO1 derivative with an in-frame deletion of the PA5517 coding sequence	This work
ΔPA4029 ΔPA4011	ΔPA4029 derivative with an in-frame deletion of the PA4011 coding sequence	This work
Δ*arnBCA*	PAO1 derivative with a deletion of the *arnBCA* genes	Lo Sciuto and Imperi, 2018
PAO1 P*rpsA*::*arn*	PAO1 derivative in which the promoter of the *arn* operon is replaced by the promoter of the housekeeping gene *rpsA*	Lo Sciuto *et al.*, 2020
PAO1 P*rpsA*::*arn* ΔPA4011	PAO1 P*rpsA*::*arn* derivative with an in-frame deletion of the PA4011 coding sequence	This work
PAO1 P*rpsA*::*arn* ΔPA4029	PAO1 P*rpsA*::*arn* derivative with an in-frame deletion of the PA4029 coding sequence	This work

[a] References not included in the main text:

Simon R, Priefer U, Pühler A. A Broad Host Range Mobilization System for In Vivo Genetic Engineering: Transposon Mutagenesis in Gram Negative Bacteria. Nat Biotechnol 1983; 1:784-791. doi.org/10.1038/nbt1183-784.

Liss, L. New M13 host: DH5 F′ competent cells. Focus. 1987; 9-13.

Miroux B, Walker JE. Over-production of proteins in Escherichia coli: mutant hosts that allow synthesis of some membrane proteins and globular proteins at high levels. J Mol Biol. 1996; 260:289-98. doi: 10.1006/jmbi.1996.0399.

Table S5. Plasmids used in this study.

Plasmid	Relevant characteristics	Source or reference
pBluescript II	Cloning and sequencing vector; ColE1 replicon; Ap^R	Stratagene
pDM4	Suicide vector used for deletion mutagenesis in *P. aeruginosa*; *sacB, oriR6K*; Cm^R	Milton *et al.*, 1996
pDM4ΔPA1209	pDM4 derivative for the in-frame deletion of the PA1209 coding sequence	This work
pDM4ΔPA2752	pDM4 derivative for the in-frame deletion of the PA2752 coding sequence	This work
pDM4ΔPA4011	pDM4 derivative for the in-frame deletion of the PA4011 coding sequence	This work
pDM4ΔPA4029	pDM4 derivative for the in-frame deletion of the PA4029 coding sequence	This work
pDM4ΔPA5244	pDM4 derivative for the in-frame deletion of the PA5244 coding sequence	This work
pDM4ΔPA5517	pDM4 derivative for the in-frame deletion of the PA5517 coding sequence	This work
pME6032	IPTG-inducible expression vector; $lacI^Q$, Tc^R	Heeb *et al.*, 2002
pMEPA4011	pME6032 derivative containing the PA4011 coding sequence downstream of the IPTG-inducible promoter	This work
pMEPA4029	pME6032 derivative containing the PA4029 coding sequence downstream of the IPTG-inducible promoter	This work
pMEDUF368	pME6032 derivative containing the coding sequence of the *P. stutzeri* KC gene CXK92_RS12370 downstream of the IPTG-inducible promoter	This work
pET15b-TEV-GFP-6×His	pET15b derivative for expression of proteins with a C-terminal TEV-GFP-6×His tag	Oluwole *et al.*, 2024
pET15b-PA4029-TEV-GFP-6×His	pET15b-TEV-GFP-6×His derivative for expression of PA4029-TEV-GFP-6×His	This work

Table S6. Primers used in this study[a].

Primer	Sequence (5'-3')	Restriction site[b]	Application
PA1209_mut_UP_FW	ccgctCGAGCGCGACGGTCGTTTC	XhoI	
PA1209_mut_UP_RV	cgggaTCCAGCAGGGGTTGCAGTT	BamHI	Generation of
PA1209_mut_DOWN_FW	cgggatcCCTGAGCGACGCCCACC	BamHI	pDM4ΔPA1209
PA1209_mut_DOWN_RV	gctctAGACCGGCGAGGTGCTGG	XbaI	
PA2752_mut_UP_FW	ccgcTCGAGGTCGATCGCTTCGC	XhoI	
PA2752_mut_UP_RV	cgggatcCGTACGCGGCCCAGTCAG	BamHI	Generation of
PA2752_mut_DOWN_FW	cgggatcCCTGGCTGCGCTGGTGC	BamHI	pDM4ΔPA2752
PA2752_mut_DOWN_RV	gctctaGACAACGACGACCCCGAAC	XbaI	
PA4011_mut_UP_FW	ccgctcgaGATCAAGTCCGGCCATCCC	XhoI	
PA4011_mut_UP_RV	cggaatTCCAGGCGTTGAAGCTGTC	EcoRI	Generation of
PA4011_mut_DOWN_FW	cggaattCGCGCTACCGGCCGCTG	EcoRI	pDM4ΔPA4011
PA4011_mut_DOWN_RV	gctctaGACCATCGGCGAAGGGCG	XbaI	
PA4029_mut_UP_FW	ccGCTCGAGCACGCCCGGC	XhoI	
PA4029_mut_UP_RV	cgggaTCCATTGAGCAACCTGCGG	BamHI	Generation of
PA4029_mut_DOWN_FW	cgggatcCCGCCAAACAGGCCAAGG	BamHI	pDM4ΔPA4029
PA4029_mut_DOWN_RV	gctctaGAGCATGTCCAGCTTGTGC	XbaI	
PA5244_mut_UP_FW	ccgctcgAGCAACTGGTCGATGGACAG	XhoI	
PA5244_mut_UP_RV	cggaatTCCTGCAGGAATTGTTGGAG	EcoRI	Generation of
PA5244_mut_DOWN_FW	cggaaTTCAAGACACCGCGCGGC	EcoRI	pDM4ΔPA5244
PA5244_mut_DOWN_RV	gctctaGAGGTTCTTCGCAGCGCC	XbaI	
PA5517_mut_UP_FW	ccgctCGAGATGCCGGCGCAGG	XhoI	
PA5517_mut_UP_RV	cgggaTCCAGTCGTAGAGACGGCG	BamHI	Generation of
PA5517_mut_DOWN_FW	cgggatcCTGATGGCGGTGATGGTGG	BamHI	pDM4ΔPA5517
PA5517_mut_DOWN_RV	gctctaGACAGCTTCTGCGACCGC	XbaI	
PA4011_pME6032_FW	cggaattCAATGAGTCTCGACAGCTTC	EcoRI	Generation of
PA4011_pME6032_RV	cccctcGAGCGCCAACCCTCACAG	XhoI	pMEPA4011
PA4029_pME6032_FW	cggaatTCAATGGACTTCAACCCAATC	EcoRI	Generation of
PA4029_pME6032_RV	cccctcGAGCTCTTCGGCCACCGG	XhoI	pMEPA4029
Ps_DUF368_pME6032_FW	cggaattCATGAAGAACGCTTTATTGTTG	EcoRI	Generation of
Ps_DUF368_pME6032_RV	cccgaGCTCCCTTTCATAGACAAGTG	SacI	pMEDUF368$_{Ps}$[c]
M13_FW	GTTTTCCCAGTCACGAC		DNA sequencing
M13_RV	AACAGCTATGACCATG		from pBS
pME6032_FW	GCTCTCGGGTAACATCAAG		DNA sequencing
pME6032_RV	CGGTTCTGGCAAATATTCTG		from pME6032

[a] Unless otherwise stated, the genomic DNA of *P. aeruginosa* PAO1 was used as the template for the preparative PCRs for cloning.

[b] The restriction site used for cloning is underlined in the primer sequence.

[c] The genomic DNA of *P. stutzeri* KC was used as the template for the preparative PCR for cloning.

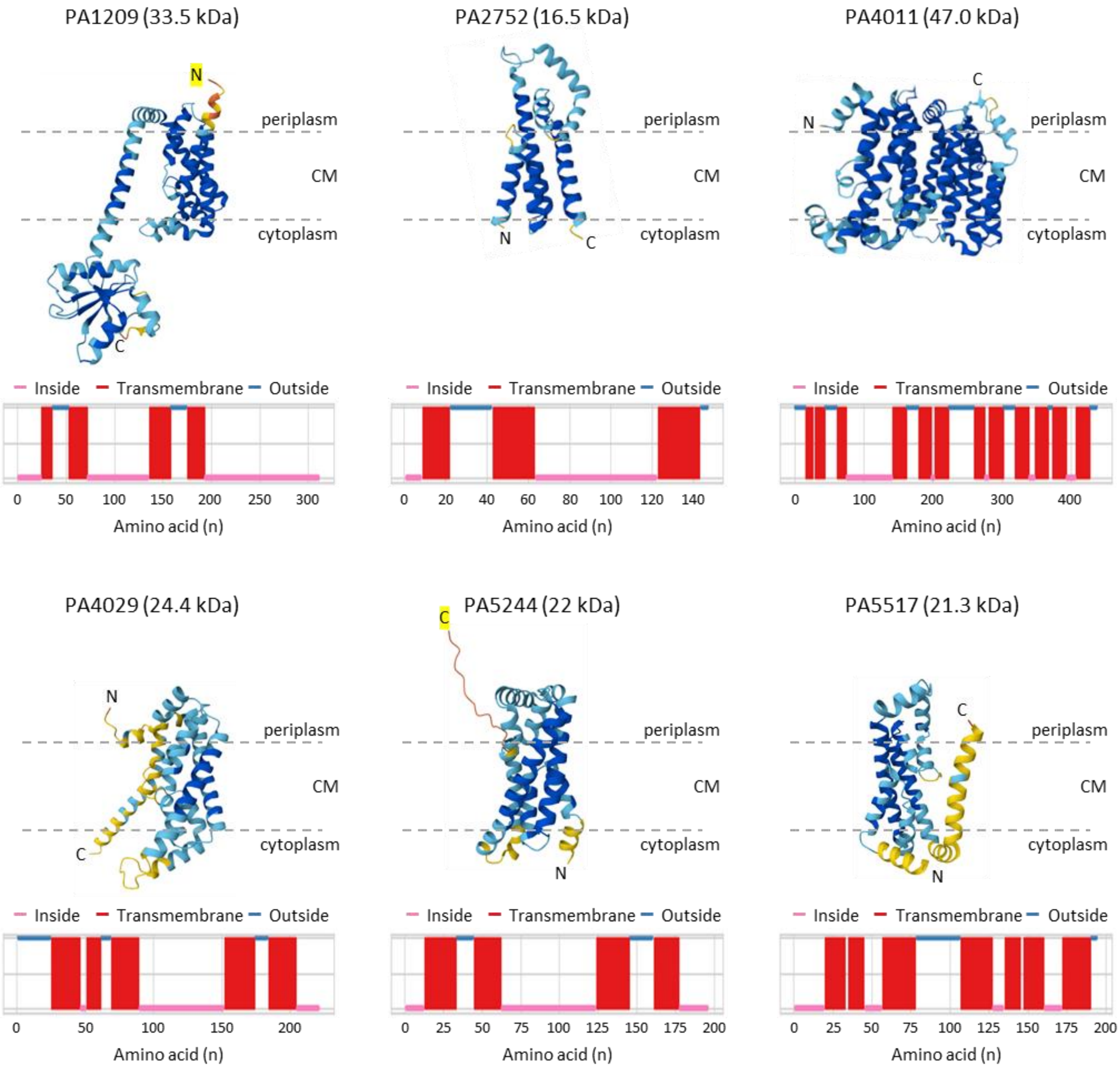

Figure S1. Three-dimensional structure (upper panels) and transmembrane helices (TMHs, lower panels) predicted for the six *P. aeruginosa* DedA-like proteins by AlphaFold and DeepTMHMM, respectively. The topology of the protein structures with respect to the cytoplasmic membrane (CM) was assumed based on the TMHs predicted by DeepTMHMM. N- or C-termini highlighted in yellow denote discrepancies between AlphaFold and TMHMM predictions.

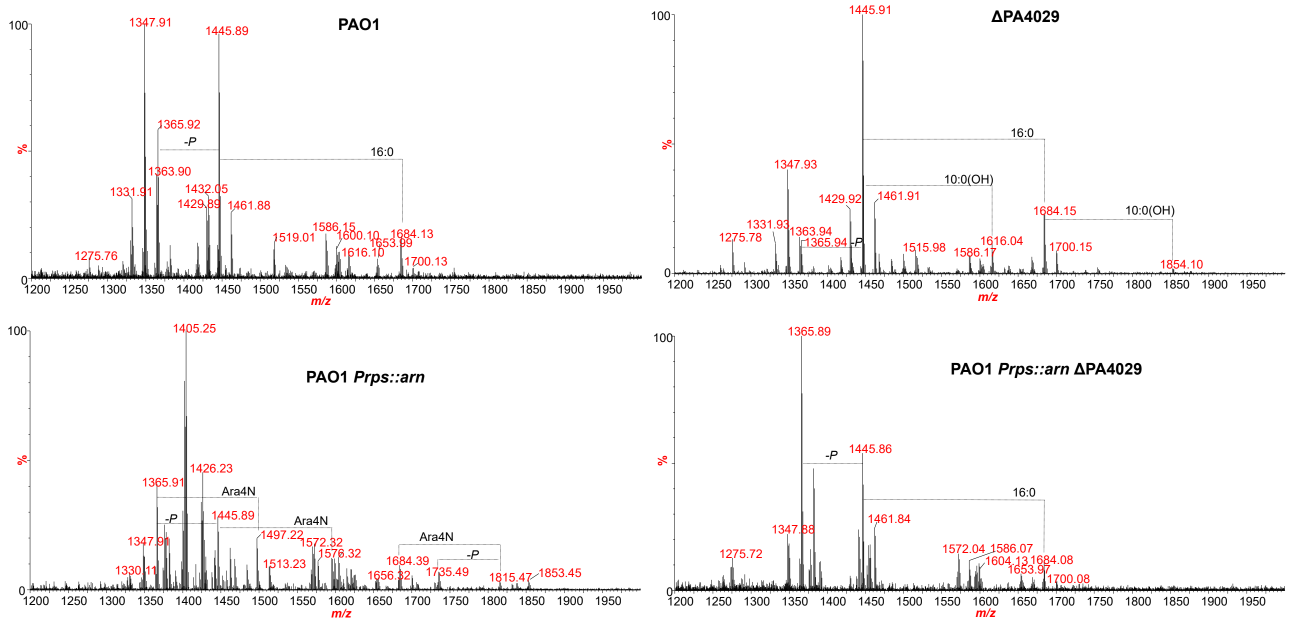

Figure S2. High-resolution, negative-ion MALDI-TOF MS spectra of lipid A from PAO1, ΔPA4029, PAO1 P*rpsA*::*arn*, and PAO1 P*rpsA*::*arn* ΔPA4029. The two main clusters of peaks are associated with *bis*-phosphorylated penta-acylated and hexa-acylated lipid A. Spectra also reveal the presence of their respective *mono*-phosphorylated forms. "-*P*" indicates the lack of a phosphate group. When present, peaks attributed to lipid A species carrying Ara4N decoration(s) have been indicated.

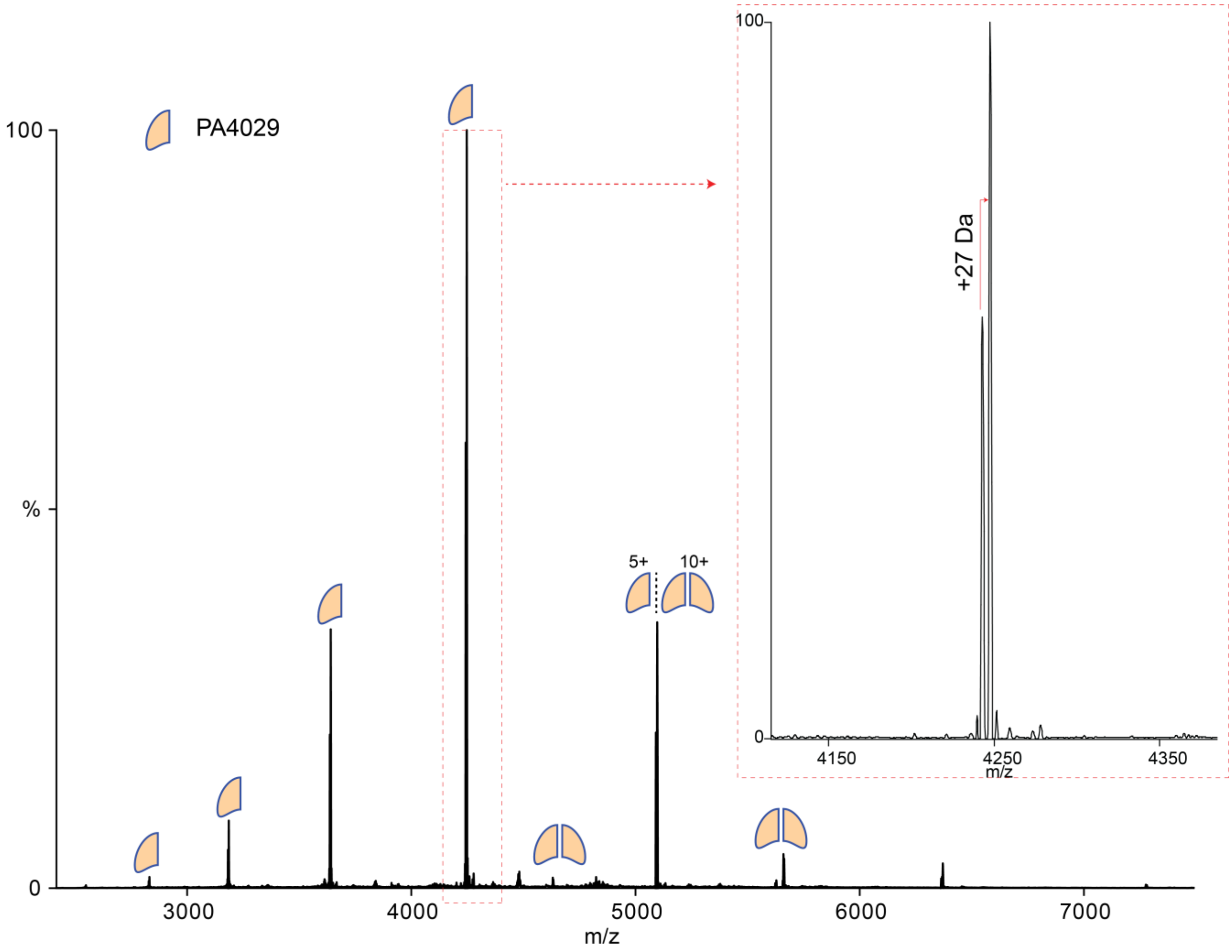

Figure S3. Purified PA4029 is in a monomer-dimer equilibrium. The mass spectrum of PA4029 shows two distinct charge-state distributions corresponding to monomers and dimers in solution. A zoom-in view of the monomeric 6+ charge state shows an addition of ~27 Da, corresponding to formylation of the N-terminal methionine.

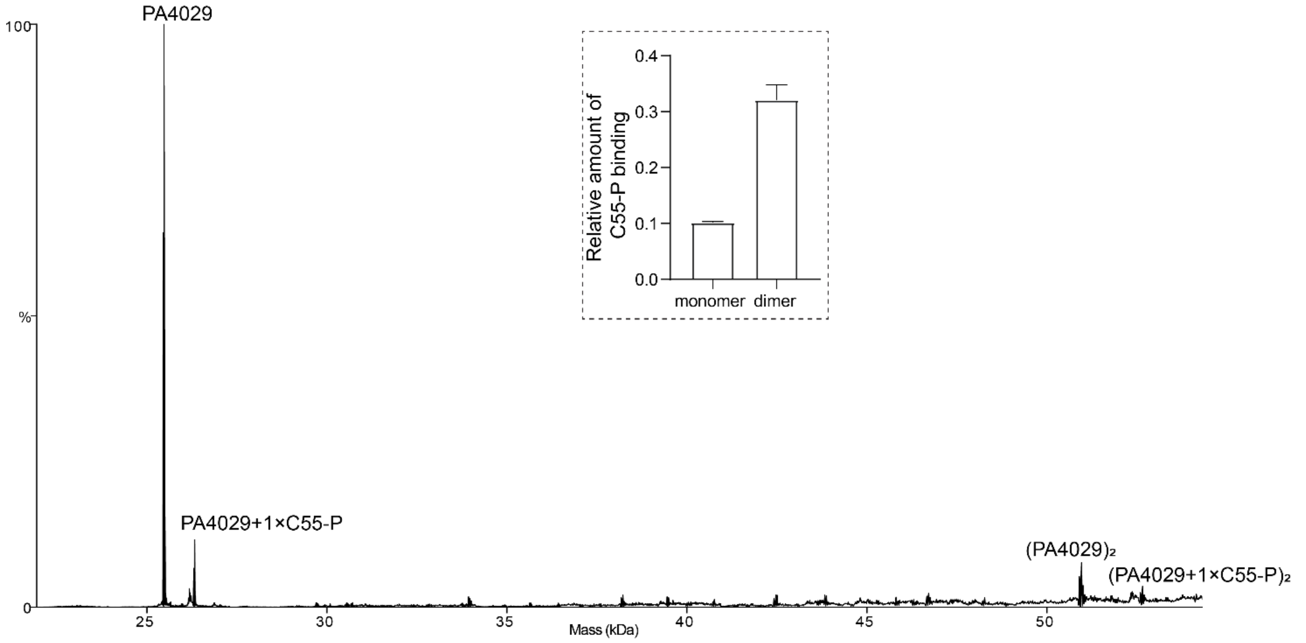

Figure S4. C55-P binds to both monomeric and dimeric PA4029. Deconvolution of the PA4029+C55-P spectrum (shown in Fig. 3) indicates that adduct peaks corresponding to C55-P binding occur with both monomeric and dimeric species. The relative amount of C55-P binding to the dimer is greater than to the monomeric species.

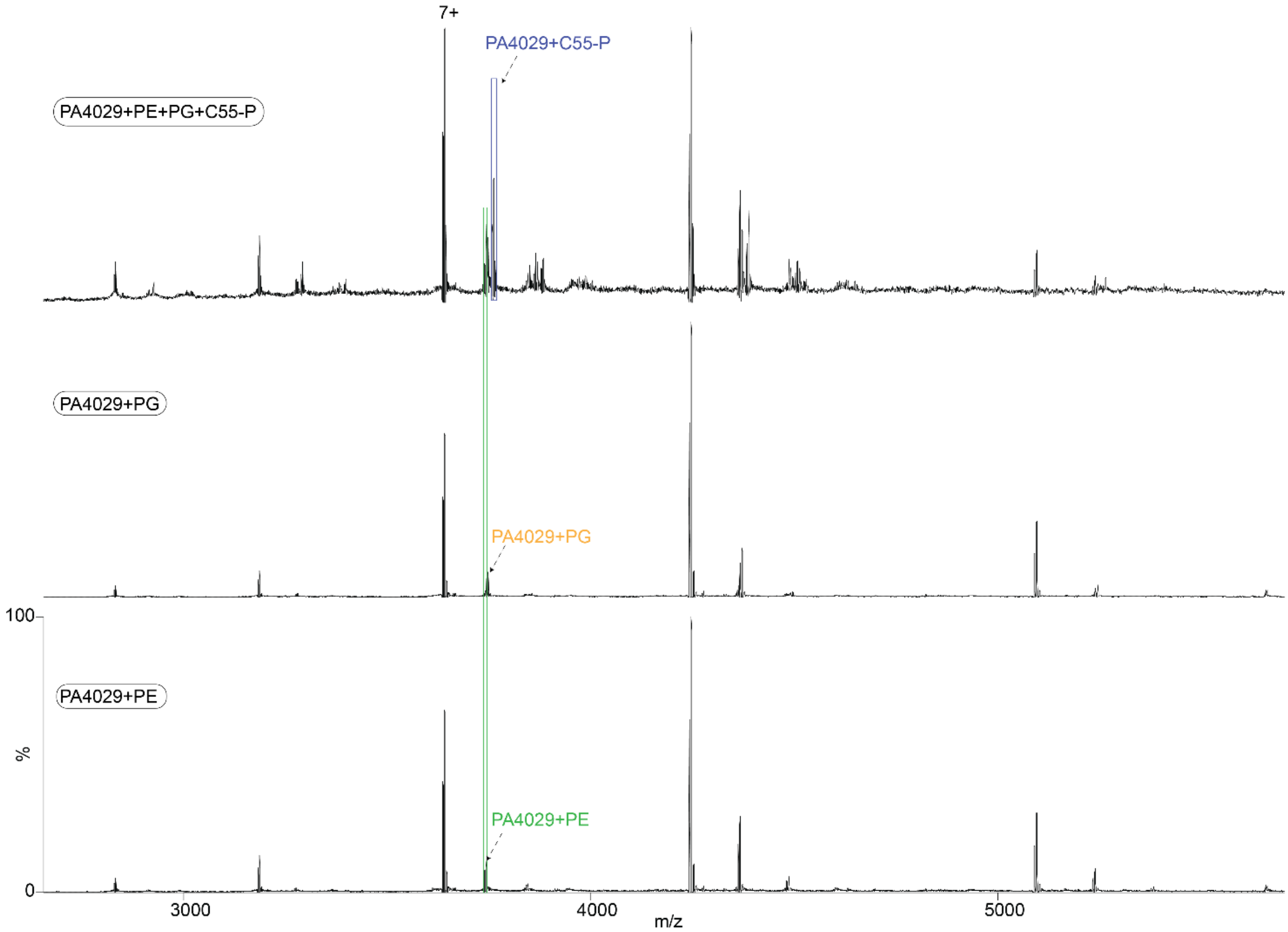

Figure S5. Binding analysis of PA4029 with phospholipids and C55-P. Mass spectra of PA4029 with PE (bottom), PG (middle) and PE+PG+C55-P (top). PE binding is highlighted in green and C55-P binding in blue.
